# Biomarkers of post-stroke cognitive impairment—a systematic literature review

**DOI:** 10.3389/fnbeh.2026.1864277

**Published:** 2026-06-22

**Authors:** Lars Schmidli, Ines Richter, Shilpa Thaliyath, Johannes Frenger, Annaelle Zietz, Johannes Teller, Ramona Schuppner, Johanna Ernst, Maria M. Gabriel, Pol Camps-Renom, John McCabe, Steffen Tiedt, Michelle C. Johansen, Mira Katan, Gerrit M. Grosse

**Affiliations:** 1 Department of Neurology and Stroke Center, University Hospital Basel, Basel, Switzerland; 2 Department of Neurology, Hannover Medical School, Hannover, Germany; 3 Department of Neurology, Hospital de la Santa Creu i Sant Pau, Biomedical Research Institute Sant Pau, Barcelona, Spain; 4 University College Dublin School of Medicine, Dublin, Ireland; 5 Stroke Service, Department of Geriatric Medicine, Mater Misericordiae University Hospital, Dublin, Ireland; 6 Institute for Stroke and Dementia Research, University Hospital, LMU Munich, Munich, Germany; 7 The Johns Hopkins University School of Medicine, Baltimore, MD, United States; 8 Department of Clinical Research, University of Basel, Basel, Switzerland

**Keywords:** biomarkers, dementia, inflammation, metabolism, neuroimaging, post-stroke cognitive impairment, small vessel disease

## Abstract

**Background:**

Post-stroke cognitive impairment (PSCI) severely affects quality of life and prognosis in stroke survivors, yet reliable predictive, diagnostic and prognostic tools remain limited. Recent advances suggest that molecular, genetic, and imaging-based biomarkers may improve prediction and management of PSCI. The purpose of this systematic review was to provide a synthesis of the current evidence on biomarkers related to PSCI, aiming to highlight knowledge gaps and future research directions.

**Methods:**

We conducted a systematic review of observational cohort, cross-sectional, case-control and interventional studies investigating biomarkers associated with PSCI and post-stroke dementia. Eligible studies enrolled patients with acute ischemic or hemorrhagic stroke and reported cognitive outcomes with biomarker assessments in blood, or other body fluids, or neuroimaging. Screening followed PRISMA guidelines. Data extraction included study design, population characteristics, biomarkers, and cognitive outcomes. Biomarkers were clustered by type for evidence synthesis.

**Results:**

Of 834 screened studies, 154 were included (99 cohorts, 19 case-control, 35 cross-sectional, 1 interventional; *n* = 114,474). The most frequently studied biomarkers were imaging biomarkers, such as infarct volume, brain atrophy, and white matter hyperintensities, which showed consistent associations with PSCI, whereas blood-based biomarkers, notably markers of inflammation (e.g., CRP, IL-6), metabolism (e.g., HbA1c, homocysteine), and neuronal damage (e.g., amyloid-β_1–42_, plasma neurofilament light chain), yielded more variable results, thereby limiting their applicability in clinical practice. Genetic markers (e.g., related to APOE ε4, MMP-9, microRNAs) showed moderate evidence of association with PSCI. We found substantial heterogeneity in timing of sampling or acquisition, cognitive assessment tools and population characteristics.

**Conclusion:**

Several body-fluid-based, genetic, and imaging biomarkers demonstrate potential for the prediction of PSCI. However, clinical utility remains constrained by study heterogeneity, lack of standardization and residual confounding. Standardized protocols for cognitive assessments and biomarker timing, as well as multi-center longitudinal studies, are essential to validate and translate these biomarkers into routine clinical care.

**Systematic review registration:**

https://www.crd.york.ac.uk/PROSPERO/view/CRD42025642710, identifier CRD42025642710.

## Graphical Abstract

**Figure:**
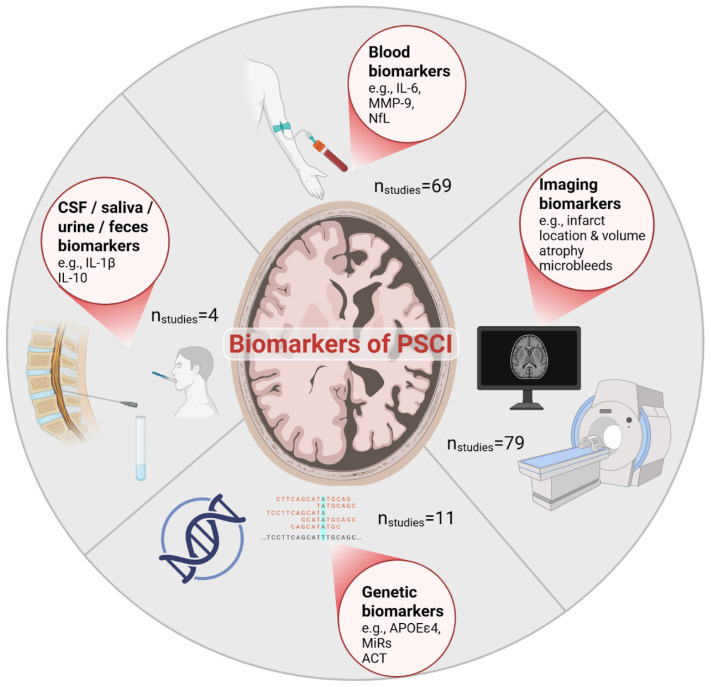

## 1 Introduction

Post-stroke cognitive impairment (PSCI) and post-stroke dementia (PSD) is one of the most common and severe consequences of stroke, profoundly impacting the quality of life and long-term prognosis of stroke survivors (El Husseini et al., 2023; Martin et al., 2025). With increasing stroke survivors, identifying those at risk for PSCI has become a clinical priority. Yet, despite the clinical significance of PSCI, reliable and efficient diagnostic and prognostic tools remain limited and PSCI is still a largely under-investigated endpoint in studies (Rost et al., 2022). Moreover, methods to investigate PSCI, including cognitive assessment and neuroimaging, offer valuable insights but often lack the sensitivity and specificity needed for early prediction to guide individualized therapy (Rost et al., 2022).

In recent years, considerable attention has focused on the potential of biomarkers, including molecular, genetic, and imaging-based indicators, to improve prediction, diagnosis, and management of PSCI (Martin et al., 2025). Blood-based biomarkers related to inflammation, metabolism, neurodegeneration, and other pathways have emerged as promising tools in research, though their clinical utility remains under investigation. At the same time, neuroimaging markers like infarct volume, cerebral atrophy, and indices of small vessel disease are being studied for their predictive value for PSCI, with ongoing work highlighting both their promise and the challenges posed by heterogeneity in study design and cognitive assessment tools.

Understanding and evaluating these biomarkers is crucial not only for early identification and prognosis, but also for unraveling the underlying molecular mechanisms of post-stroke cognitive decline and discovering novel therapeutic targets. This systematic review aims to provide a comprehensive overview of current evidence regarding biomarkers for PSCI. By summarizing the evidence of body-fluid-based, structural brain imaging, and genetic biomarkers, we seek to highlight knowledge gaps, and identify potential avenues for future approaches.

## 2 Methods

This systematic review followed PRISMA 2020 recommendations for reporting systematic reviews, and the search component was reported in accordance with the PRISMA-S extension. The review protocol was registered in PROSPERO (CRD42025642710).

### 2.1 Search strategy and information sources

In this systematic review, we aimed to identify biomarkers (blood-based, cerebrospinal-fluid-(CSF)-based, other body-fluids, structural brain imaging, genetics) associated with PSCI and post-stroke dementia. We conducted a literature search on MEDLINE PubMed between 15 January and 10 June 2024, using the following search strategy: (“post-stroke cognitive impairment” OR “post-stroke dementia” OR “post-stroke cognitive impairment and dementia” OR “PSCI” OR “PSCD” OR “PSCID” OR “vascular cognitive impairment”) AND (“biomarker” OR “stroke etiology” OR “stroke pattern” OR “brain atrophy” OR “atrophy” OR “brain damage” OR “infarct pattern” OR “vascular territory” OR “territory” OR “pattern” OR “lobar” OR “deep” OR “non-lobar” OR “imaging” OR “blood” OR “CSF” OR “cerebrospinal fluid” OR “polymorphism” OR “snp” OR “genetic variant”) AND (“stroke” OR “ischemic stroke” OR “ischaemic stroke” OR “hemorrhagic stroke” OR “haemorrhagic stroke” OR “brain hemorrhage” OR “brain hemorrhage”). We included observational cohort, cross-sectional case-control and prospective intervention studies. Same strategy was applied using EMBASE and results were compared with those retrieved from MEDLINE PubMed.

### 2.2 Eligibility criteria

We included studies if they met the following criteria: (1) patients had experienced clinical acute ischemic or hemorrhagic stroke; (2) cognitive outcomes, including PSCI or post-stroke dementia were reported as the primary endpoint; and (3) biomarkers were measured in blood, cerebrospinal fluid (CSF), other body fluids (e.g., urine), or by neuroimaging as predictor or exposure variables. We excluded studies based on the following criteria: (1) inappropriate study designs such as meta-analyses, reviews, case reports (2) premorbid dementia diseases such as Alzheimer’s or vascular dementia; (3) more than 50% transient ischemic attacks (TIAs) in the stroke cohort; (4) not written in English; (5) studies investigating chronic cerebrovascular disease (e.g., cerebral amyloid angiopathy, Cerebral Autosomal Dominant Arteriopathy with Subcortical Infarcts and Leukoencephalopathy (CADASIL)) but not acute stroke; or inadequate control group.

### 2.3 Selection process

During the screening process from 15 January 2024, until 10 June 2024, we identified a total of 834 studies from both databases. These studies were screened for eligibility based on the predefined inclusion and exclusion criteria. The screening process followed a two-step approach: first, abstracts were reviewed by L.S. to exclude inappropriate studies; second, full-text articles of the remaining studies were examined to extract relevant data. Studies were independently screened by a second researcher (G.M.G.) to ensure eligibility. We included 154 studies in the final analysis.

### 2.4 Data extraction

From the included studies, we extracted the following data: PubMed ID; study design; sample sizes including outcome group (number of participants with cognitive decline or post-stroke dementia), control group (number of control participants), sex distribution, mean (or median) age, and ethnicity; whether the study was part of a consortium and the consortium name; time intervals between stroke and biomarker measurement or imaging, time intervals between stroke and cognitive assessment, and whether multiple cognitive assessments had been conducted over time; cognitive testing tools used [e.g., Montreal Cognitive Assessment (MoCA), Mini-Mental State Examination (MMSE)]; stroke characteristics, including the percentage of participants with transient ischemic attacks (TIAs), prior strokes, stroke etiology (large artery atherosclerosis, cardioembolism, small vessel disease, cryptogenic/undetermined, other), stroke pattern (embolic, territorial, lacunar), and type of hemorrhagic stroke (lobar, non-lobar); secondary atrophy (yes/no), and if yes, where; sources of the biomarkers assessed (blood, CSF, other, imaging), single nucleotide polymorphism or hereditary markers as genetic biomarkers (yes/no), and the name of the biomarker; and the main finding of the study relevant to our research question, effect sizes with confidence intervals and *p*-values.

Due to the heterogeneity of data and study designs, we did not perform a meta-analysis but instead clustered the biomarkers of interest according to type (e.g., blood-based, CSF-based, imaging, etc.). We summarized the evidence available for different clusters of biomarkers with regard to outcomes. We conceptualized biomarkers according to their temporal relationship to stroke and cognitive outcomes. Predictive/prognostic biomarkers were defined as any biomarker measured at or after the index stroke (acute, subacute or chronic phase) and examined for its association with subsequent PSCI or post-stroke dementia at a later time point. Diagnostic biomarkers were defined as markers measured concurrently with cognitive assessment and evaluated for their ability to distinguish patients with PSCI or post-stroke dementia from cognitively unaffected stroke survivors.

### 2.5 Risk of bias assessment

The Quality In Prognosis Studies (QUIPS) tool was used to assess the risk of bias across all included studies (QUIPS Tool, 2025). QUIPS domains (study participation, study attrition, prognostic factor measurement, outcome measurement, study confounding, and statistical analysis/reporting) were rated as low, moderate or high risk of bias, and summarized graphically.

### 2.6 Synthesis methods

Due to substantial clinical, methodological and statistical heterogeneity across studies, a quantitative meta-analysis was not feasible. Sources of heterogeneity included: variability in timing of biomarker sampling and imaging acquisition (acute vs. subacute vs. chronic), diversity of cognitive outcome definitions and measurement tools, wide variation in follow-up duration, and non-uniform adjustment for key confounders such as age, education, premorbid cognition and vascular risk factors. In line with the SWiM guidance for reviews without meta-analysis, we therefore conducted a structured narrative synthesis. For evidence synthesis, we first grouped studies by biomarker category (body-fluid, genetic/molecular, imaging, other) and, within each category, by related pathophysiological domains. Within these groups, we distinguished cohorts by stroke type, presence of TIA, and follow-up duration, as reported in the tables. We then summarized study-level characteristics and effect estimates in structured tables, recording direction and statistical significance of associations.

## 3 Results

**FIGURE 1:**
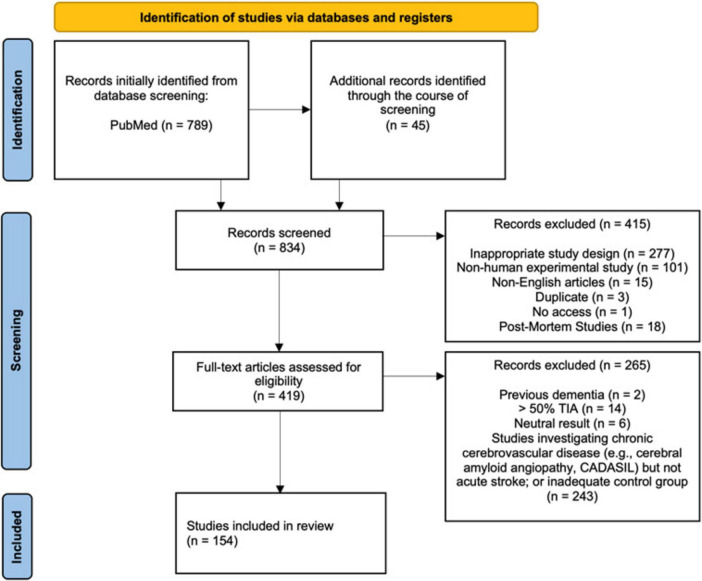
PRISMA flow diagram. Overview on study selection.

Following a full-text review, 154 studies were included in the final analysis, comprising 99 cohort studies, 19 case-control studies, 35 cross-sectional studies, and one interventional study. The study selection process is shown in the PRISMA flow diagram (Figure 1).

Body-fluid biomarkers reported in the included studies are shown in Supplementary Table 1, genetic/molecular biomarkers in Supplementary Table 2 and imaging biomarkers in Supplementary Table 3. Studies that could not be clearly categorized were listed as “others” in Supplementary Table 4.

The included studies were published between 1996 and 2024. Sample sizes ranged from 17 to 17,729, with an overall total of 114,474 individuals considered in this analysis. Participant ancestry in the 154 included studies was predominantly Asian [*n* (study) = 101] and Caucasian (*n* = 53), with some studies including multiple ancestry groups. A minority of studies included Arabic (*n* = 3), Black (*n* = 2), or Indian (*n* = 2) populations.

Ischemic stroke was investigated in 124 studies, hemorrhagic stroke in three studies, and both stroke types in a mixed population in 27 studies. Ten studies included participants who had experienced a transient ischemic attack (TIA), with the proportion of TIA cases in these cohorts ranging from 13% to 33%. A total of 60 studies only included participants who had experienced their first-ever stroke. The most frequently used cognitive assessments were the MoCA and the MMSE, with several studies employing both tools. The timing of cognitive assessment after stroke varied, ranging from a few days after hospital admission to several years post-stroke. In certain studies, cognitive assessments were conducted at multiple follow-up time points.

The identified biomarkers were grouped according to the biological source and method of assessment:

(1) Body-fluid biomarkers (blood, CSF, urine, saliva, stool or combination thereof): A total of 73 studies investigated circulating biomarkers, including markers of inflammation, metabolism and neuronal damage, as well as cellular and protein biomarkers. Circulating biomarkers were predominantly blood-based. In contrast, only 1 study investigated biomarkers in CSF, 1 in urine, 1 in stool, and 1 in saliva. Glucose metabolism-related markers were the most frequently examined blood-based biomarkers associated with PSCI, with blood glucose (diabetes mellitus) reported in eight studies, HbA1c in three, and related variables such as prediabetes, diabetes duration, and history of diabetes each cited once. Other metabolic biomarkers such as homocysteine (Hcy) were frequently reported, with Hcy appearing in eight studies, and one study examining it specifically in combination with hypertension. Additional metabolic markers included low folate (two studies) and various lipid-related biomarkers, such as LDL (two studies), hyperlipidemia, low triglycerides (TG), TG/HDL-C ratio, and HDL-C (each reported once). Uric acid (UA) was also reported in two studies. Anemia and mean corpuscular volume (MCV) as biomarkers were each mentioned in two studies. Protein biomarkers such as amyloid-beta 42 (Aβ_1–42_/Aβ_42_) and neurodegeneration-related damage markers like plasma neurofilament light chain (pNfL) were each reported in two studies. Interestingly, one study identified low Aβ_1–42_ levels as a potential biomarker for PSCI, whereas the other discussed elevated Aβ_42_ levels in the same context.

Supplementary Table 1 provides an overview on all identified circulating biomarkers and their association with post-stroke cognitive outcomes.

(2) Genetic biomarkers: Genetic biomarkers were assessed in 11 studies, with a focus on single nucleotide polymorphisms (SNPs), gene expression, and microRNAs. The apolipoprotein E ε4 allele (*APOE e4*) was identified in three studies as being associated with an increased risk of PSCI. APOE ε4 status was defined as carriers (≥1 ε4 allele) vs non-carriers in all these three studies.

Supplementary Table 2 provides a summary of all identified genetic and molecular biomarkers and their association with post-stroke cognitive outcomes.

(3) Imaging biomarkers: Imaging-derived biomarkers were evaluated in 79 studies. These included parameters such as atrophy, white matter hyperintensities, infarct volume, stroke location, and microbleeds. Certain markers were reported more than once; for example, regarding stroke location, *thalamic infarctions or lesions* were the most frequently described, appearing in five studies and, regarding primary atrophy, *medial temporal (lobe) atrophy* (*MTA/MTLA)* were reported eight times. White matter alterations, including white matter hyperintensities, lesions, and diffusion abnormalities, have been frequently reported as potential biomarkers for PSCI, with a total of 41 studies supporting this association. Supplementary Table 3 summarizes the main imaging findings and the predictive value of radiological features.

(4) Others: 12 studies, summarized in Supplementary Table 4, investigated biomarkers that could not be clearly assigned to the categories listed above. These mostly involved imaging scores, with the *modified small vessel disease (SVD) score* reported twice, while the *SVD score* was reported once.

In Supplementary Table 5, the biomarkers for each category are listed, with each biomarker included only once, resulting in a total of n distinct biomarkers per category. Supplementary Table 6 presents the different imaging biomarkers for each category.

**FIGURE 2:**
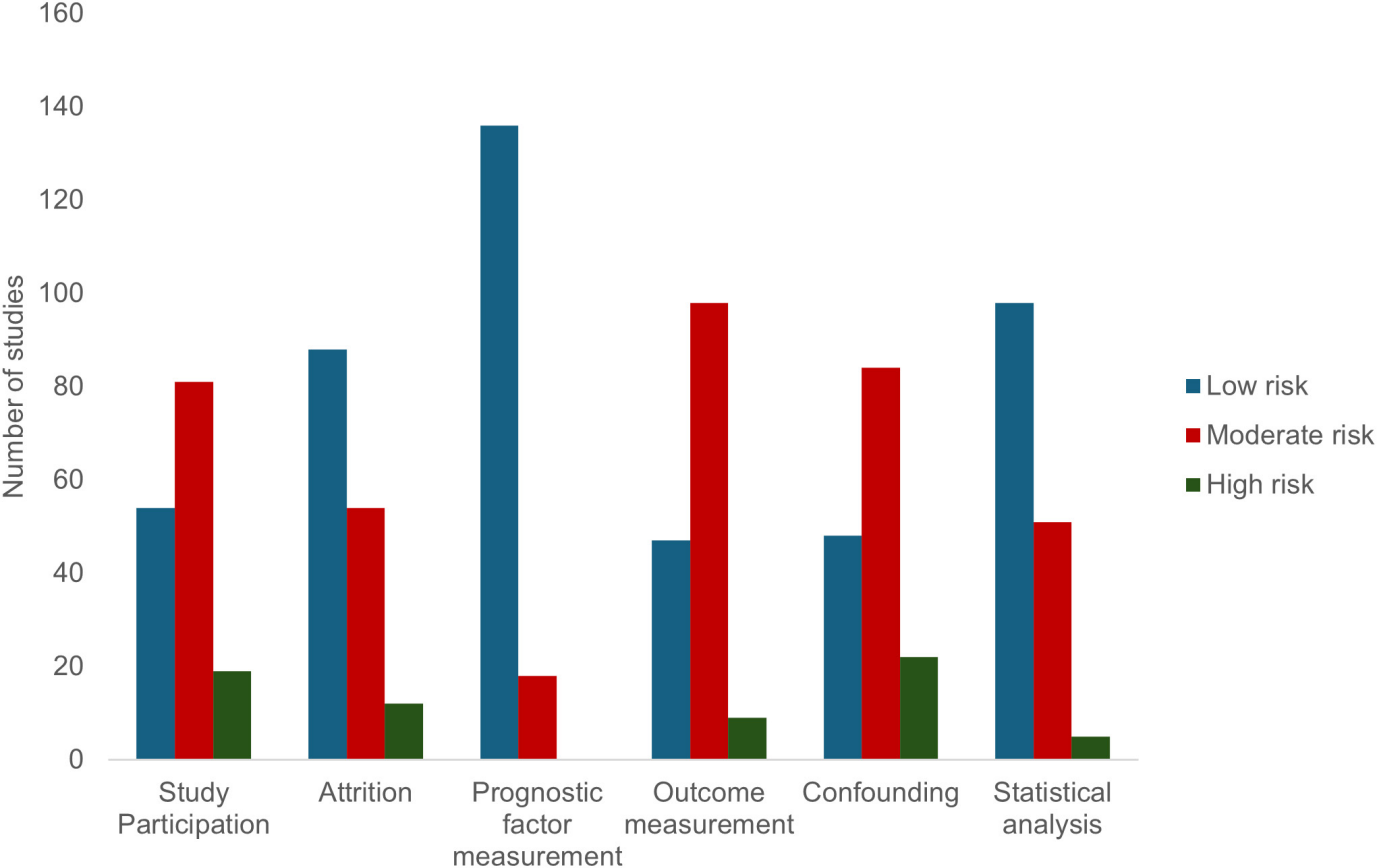
Risk-of-bias assessment. Assessment of risks of bias applying the Quality in Prognostic Studies (QUIPS) tool.

(5) Risk of bias analysis: Using the QUIPS tool, the domains most frequently associated with potential bias were study attrition, confounding factors, and outcome measurement. Overall, the risk for potential biases across all studies was low to moderate (Figure 2). Further evaluation identified recurring limitations, such as varying time points for outcome assessment, insufficient consideration of the reasons for dropouts, and potentially insufficient deconfounding.

## 4 Discussion

This systematic review summarizes the current evidence on biomarkers for PSCI, revealing important insights as well as substantial heterogeneity in study designs, populations, and methodologies that limit the strength of conclusions.

### 4.1 Body-fluid biomarkers

Body-fluid biomarkers offer an accessible approach to obtain potentially valuable diagnostic and prognostic information at the molecular or cellular level.

In the subgroup of inflammation and oxidative stress, biomarkers such as CRP, IL-6, rheumatoid factor (RF), Malondialdehyd (MDA), and 8-Hydroxydesoxyguanosin (8-OHdG) were identified, consistent with findings from previous reviews (Kim et al., 2022; Ma et al., 2024; Mijajlović et al., 2017; Zhang and Bi, 2020). There is a broad body of evidence showing that proinflammatory cytokines worsen neuroinflammation and accelerate cognitive decline by secondary brain tissue damage (Shang et al., 2022). Elevated levels of peripheral white blood cells (WBCs), particularly neutrophils, contribute to PSCI by promoting oxidative stress, endothelial injury, and brain blood barrier (BBB) disruption, possibly through matrix metalloproteinase-9 (MMP-9) activity (Shan et al., 2021; Ye et al., 2022). The MMP-9 rs3918242 polymorphism (TC- and CC-genotype) and elevated serum MMP-9 levels are associated with PSCI. This association likely involves BBB disruption, neuroinflammation, and white matter damage, as supported by both clinical and preclinical evidence (Zhao et al., 2022; Zhu et al., 2019). Higher neutrophil-to-lymphocyte ratios (NLRs) reflecting systemic inflammation correlate with greater hippocampal damage and impaired neurogenesis in PSCI (Shang et al., 2022). While biomarkers such as MMP-9, NLR, and total WBC count show potential diagnostic value for PSCI, their underlying pathomechanisms remain incompletely understood.

In contrast, a recent prospective cohort study found that CRP, WBC, and NLR levels, which are frequently elevated in stroke patients at the time of hospital admission, did not predict PSCI at the time of the first cognitive assessment (7–10 days post-stroke), nor at the 6-month follow-up (Milosevich et al., 2024). Future studies should therefore aim to characterize inflammatory responses more precisely and investigate the long-term cognitive effects of post-stroke inflammatory responses. It moreover remains unknown whether emerging anti-inflammatory approaches for secondary stroke prevention, like applying Colchicine or specific drugs targeting the IL-1β-IL-6-CRP axis, could also show beneficial properties in preventing PSCI (Kelly et al., 2024).

Several studies have demonstrated a significant association between metabolic disease, most importantly diabetes mellitus, and PSCI, which is in line with previous findings (Lee et al., 2023; Lo et al., 2020; Wang J. et al., 2024). HbA1c is considered a common indicator linked to the risk of PSCI (Lee et al., 2023; Ma et al., 2022). In addition to long-term blood glucose levels, short-term fluctuations, measured by indices such as mean absolute glucose (MAG) and the glucose gap, have been linked to a higher risk of PSCI three months after stroke, even in non-diabetic patients (Lee et al., 2023). Recent large-scale studies confirm and expand these findings: several glycemic variability markers, including mean amplitude of glycemic excursion (MAGE), mean postprandial blood glucose (MPBG), and the stress hyperglycemia ratio (SHR), were significantly associated with cognitive decline in both the acute and subacute phases of ischemic stroke (Zhou et al., 2024).

While the association between diabetes and PSCI is well-established, the effects of different glucose-lowering treatments on cognitive outcomes remain less clear. While metformin has been associated with both reduced and increased risks of Alzheimer’s disease in various studies, SGLT2 inhibitors show promising cognitive benefits, potentially through anti-inflammatory effects and reduced cerebrovascular risk (Lee et al., 2023). In contrast, insulin use has been linked to an increased risk of dementia, possibly due to hypoglycemia (Lee et al., 2023). These controversial findings underscore the need for further research on optimal antidiabetic treatment strategies to prevent PSCI and dementia, for example, by differentiating between ischemic and hemorrhagic stroke (Lee et al., 2023).

Some studies report higher triglycerides or related indices, such as the triglyceride-glucose (TyG) index and the TG/HDL-C ratio, as risk factors for PSCI, while others observe an inverse association (Cheng et al., 2022, 2024). A large cohort study found that lower triglyceride levels were linked to increased post-stroke dementia risk, especially in older individuals and when triglycerides were within guideline targets. This “triglyceride paradox” may reflect confounding factors like malnutrition or frailty, which often coincide with low triglycerides (Yang et al., 2022).

The role of Aβ_1–42_ as a biomarker for PSCI remains complex. Some studies report elevated plasma Aβ_1–42_ in PSCI patients, suggesting that the interplay between ischemia, amyloid accumulation, and inflammation could lead to cognitive decline following AIS (Huang et al., 2021; Tang et al., 2018). In contrast, other studies have reported reduced circulating Aβ_1–42_ levels in association with PSCI, potentially reflecting increased cerebral deposition or impaired peripheral clearance (Chi et al., 2019; Mao et al., 2020). Timing of biomarker measurement may at least partly explain these discrepancies. A study showing elevated Aβ_1–42_ levels measured biomarkers over 80 months post-stroke (Tang et al., 2018), while those reporting reduced levels collected samples within the acute phase (Mao et al., 2020), suggesting a dynamic post-stroke trajectory and highlighting the need for standardized time points in biomarker assessment to ensure comparability across studies.

Alzheimer’s pathology, vascular cognitive impairment (VCI), and PSCI, often coexist and complicate diagnosis. Ischemic stroke may trigger or accelerate AD-related neurodegeneration, with chronic hypoperfusion, such as from carotid artery stenosis, linked to increased Aβ deposition. Neuroinflammation also contributes by promoting white matter degeneration. These interacting mechanisms suggest a continuum rather than distinct pathologies, emphasizing the need to consider their overlap in understanding and managing PSCI (Thiel et al., 2014).

### 4.2 Genetic and molecular biomarkers

Identifying genetic associations with PSCI may not only improve our understanding of the molecular pathways but could also offer the opportunity to identify novel drug targets and select patients who are especially susceptible (Montaner et al., 2020).

Consistent with its established role in Alzheimer’s disease, the APOE ε4 allele is associated with pre- and post-stroke cognitive decline (Pendlebury et al., 2020). Biologically, ε4 promotes synaptic loss, amyloid deposition, and impaired neuronal repair, thereby exacerbating cognitive decline (Ballard et al., 2004; Wagle et al., 2009). A population-based longitudinal study supports independent effects of stroke and APOE ε4 on cognitive decline, with no evidence for a synergistic interaction. APOE ε4 was primarily associated with decline in information processing speed, whereas stroke and APOE ε4 appeared to influence cognition through distinct pathogenic pathways (Dik et al., 2000).

Circulating microRNAs undergo dynamic changes after stroke and may reflect systemic and neuroinflammatory responses to ischemic brain injury (Montaner et al., 2020). MicroRNAs may serve as promising biomarkers for PSCI due to their stability in serum, consistent expression across individuals, and strong correlation with cognitive function (Huang et al., 2016; Wang et al., 2020). Different microRNAs such as miR-132, miR-let-7i, miR-21, miR-200b, and miR-511-3p have also been associated with PSCI, likely involving different pathomechanisms. For example, miR-511-3p is reported to contribute to atherosclerosis development and, through modulation of neuronal differentiation, to vascular remodeling and neurodegenerative processes, whereas miR-21 plays a neuro-protective role by upregulating the anti-apoptotic protein Bcl2 (Wang et al., 2020; Wang T. et al., 2024; Yuan et al., 2022)

### 4.3 Imaging biomarkers of structural brain changes

Imaging biomarkers represent the most frequently investigated type in this systematic review. Given the wide range and increasing complexity of neuroimaging biomarkers in PSCI, this review focuses on markers of structural brain changes, while recognizing that additional functional and network-based imaging markers may be of growing relevance and were not comprehensively addressed.

Strategic and region-specific infarcts have been consistently linked to PSCI. For example lesions involving the thalamus and basal ganglia (notably the caudate and globus pallidus) as well as classically strategic sites such as the angular gyrus or hippocampus were associated with increased risk for PSCI (Chaudhari et al., 2014; Firbank et al., 2012; Weaver et al., 2021; Zhang et al., 2022). Larger infarct volumes or lesion sizes also proved as relevant structural markers (Hobden et al., 2023; Ma et al., 2023). Stroke laterality, particularly involvement of the dominant hemisphere, was correlated with worse cognitive outcomes (Gallucci et al., 2024; Gong et al., 2020; Gu et al., 2022; Schellhorn et al., 2021). Furthermore, preexisting structural brain abnormalities related to cerebral small vessel disease, including lesions such as microbleeds, lacunar infarcts and white matter changes are major contributors to cognitive impairment or dementia after stroke (Chi et al., 2020; Jaime Garcia et al., 2025; McHutchison et al., 2019; Mijajlović et al., 2017; Pavlovic et al., 2014; Rost et al., 2022; Wu et al., 2020; Yatawara et al., 2020).

White matter integrity is another evolving biomarker for PSCI. Diffusion tensor imaging (DTI) consistently shows differences in microstructural damage between cognitively healthy individuals to those with mild cognitive impairment, with reductions in fractional anisotropy (FA) and increases in mean diffusivity (MD) closely linked to deficits in processing speed, executive function, and memory (Du et al., 2021; Egle et al., 2022).

Diffusion tensor imaging measures outperform conventional MRI markers such as white matter hyperintensity lesion load in detecting executive dysfunction (Lawrence et al., 2013). In addition, microstructural abnormalities along white matter tracts remote from lacunar infarcts, undetectable on standard MRI, are associated with worse cognitive outcomes (Reijmer et al., 2013).

In addition to standard DTI metrics (FA/MD) used to assess microstructural white matter damage in CVSD, the whole-brain diffusion tensor image segmentation metric (DSEG-θ) provides a single, comprehensive measure of microstructural changes across the cerebrum. Multivariable models identify DSEG-θ as a stable and significant predictor of cognitive change, often outperforming traditional MRI measures. Together, these findings position DSEG-θ as a practical and sensitive single-modality biomarker for monitoring microstructural brain damage and progression in SVD (Williams et al., 2017).

### 4.4 Variability in definitions of PSCI and cognitive assessment tools

A primary challenge across the included studies was the variability in how PSCI was defined, a limitation reported in previous studies (Mijajlović et al., 2017). Most studies relied on widely used cognitive assessment tools such as the MMSE and MoCA. The thresholds for cognitive impairment and the timing of measurement differed considerably. For instance, some studies used cutoff values such as MoCA < 23 at 1 year after stroke (Chi et al., 2020), while others used MoCA < 26 at 7 days, 1 and 3 months post-stroke (Jiang et al., 2014). These different cutoff values are contributing to inconsistencies in reporting the prevalence and severity of PSCI. Next to MMSE or MoCA, other tools for defining PSCI were used, such as the DSM-IIIR criteria (Chen et al., 2016) or the NINDS-AIREN criteria (Tang et al., 2018).

The timing of cognitive assessments post-stroke varies considerably across studies, ranging from a few days to several months or even years after the event. This variability is critical, as cognitive testing too early after stroke may fail to detect later-onset cognitive decline, early testing may include only less severely affected patients at onset and acute-phase assessments may underestimate cognitive function due to transient impairments that can recover over time (Mijajlović et al., 2017). Longitudinal studies demonstrate that cognitive performance after stroke can fluctuate over time, with some individuals showing improvement and others experiencing decline, highlighting the importance of repeated assessments to accurately capture the evolving nature of post-stroke cognitive outcomes (Oh et al., 2018; Tang et al., 2011; Williamson et al., 2008).

Several studies did not account for pre-stroke cognitive status, making it difficult to distinguish between pre-existing cognitive impairment and new-onset deficits following stroke. Given that stroke predominantly affects older adults, who are more likely to have undiagnosed cognitive decline prior to the event, assessing pre-stroke cognitive function is essential for accurately identifying post-stroke cognitive changes (Hénon et al., 1997; Mijajlović et al., 2017; Rost et al., 2021).

Notably, modest effect sizes, highlighting the difficulty in identifying reliable biomarkers for PSCI. Many significant findings in crude analyses were affected by confounding due to demographic, clinical, and vascular risk factors, highlighting the importance of thorough multivariable several studies reported associations between biomarkers and cognitive outcomes with very adjustments (Rezaei et al., 2016).

In terms of study design, our review identified an under-representation of female patients with 39% and a notable representation of Asian populations, limiting the generalizability of findings to the broader population. Further, hemorrhagic stroke was under-represented, as only 19% of the 154 studies included patients with hemorrhagic stroke. Consequently, the biomarkers identified in this review should be interpreted with caution for hemorrhagic stroke populations.

The recently presented 5-year results of the german center for neurological diseases (DZNE) mechanisms of dementia after stroke (DEMDAS) study which was published after the end of our systematic literature search, highlight the value of large prospective multicenter cohort studies in identifying key risk factors for the development of PSCI (Filler et al., 2025). Notably, metabolic syndrome is emerging as a significant and potentially modifiable contributor (Filler et al., 2025). These findings complement previous evidence, including one study from the DEMDAS consortium, which was included in our systematic review and identified high-sensitivity cardiac troponin T (hs-cTnT) as a potential biomarker for PSCI (von Rennenberg et al., 2024). As DEMDAS continues to provide high-quality longitudinal data, it will help to validate or refute existing hypotheses, such as those related to stroke-heart syndrome and autonomic dysfunction, and may uncover additional novel biomarkers. More studies of this kind will be essential in the future to deepen our pathophysiological understanding and improve prevention of cognitive decline after stroke.

### 4.5 Clinical implications and future priorities

Based on the number of studies, consistency of direction, and adjustment for confounders, the overall evidence can be broadly categorized as strong for structural imaging biomarkers, moderate for a subset of blood-based markers (e.g., glycemic indices, some inflammatory markers), and exploratory to emerging for most genetic markers.

While imaging biomarkers showed relatively consistent associations with PSCI across studies, findings for blood-based biomarkers were considerably more heterogeneous. In particular, infarct volume, brain atrophy, and white matter hyperintensities emerged as imaging markers consistently linked to PSCI and may therefore have potential clinical utility for identifying patients at increased risk. As these features are commonly available from routine stroke imaging, their systematic assessment during clinical work-up could help identifing patients at risk for PSCI and therefore may benefit from longitudinal monitoring. In contrast, findings for blood-based biomarkers showed more heterogenous results, limiting their applicability in routine clinical application at this stage, while genetic biomarkers showed overall moderate evidence regarding their association with PSCI. Still, some of these markers may reflect potentially modifiable mechanisms. Further longitudinal studies are needed to better understand the underlying pathways and to determine whether these biomarkers can eventually be integrated into clinical practice.

## 5 Limitations

This systematic review has limitations, especially concerning selection of studies. While a holistic representation of all studies performed in the field, especially regarding neuroimaging, is ambitious, we aimed to provide an overview of the current state of the art to evaluate potential future directions and to overcome limitations in study designs. Moreover, due to the heterogeneity of studies, we did not perfom a meta-analysis but decided to provide a descriptive synthesis of evidence. A further source of heterogeneity relates to the inclusion of patients with TIA in some cohorts. Because TIA is associated with nor or lower lesion burden and different risk profiles for subsequent cognitive decline than stroke, combining stroke and TIA may attenuate or distort biomarker–outcome associations. We attempted to mitigate this by excluding studies in which TIAs constituted more than half of the cohort and by clearly flagging mixed studies in the tables. Nonetheless, residual contamination by TIA cases may have contributed to variability in effect estimates for some biomarkers and should be borne in mind when interpreting the findings. Finally, despite the systematic approach including two established databases, it is possible that some relevant studies within the current evidence base were not considered due to the predefined search strategy and in-/exclusion criteria.

## 6 Conclusion

Blood-based biomarkers, including those related to inflammation and metabolic pathways emerged as some of the most frequently studied in association with PSCI. However, the clinical utility of these biomarkers remains uncertain, as many were identified in single studies or had limited predictive power. Likewise, neuroimaging biomarkers, including infarct volume and brain atrophy, also showed potential but were not consistently associated with cognitive outcomes.

To ensure more generalizable and comparable results across studies, future research should aim to establish standardized protocols for the assessment of PSCI. This includes the consistent use of validated cognitive measurement tools, systematic consideration of pre-stroke cognitive status, and clearly defined time points for cognitive testing, ideally incorporating follow-up assessments to capture the dynamic trajectory of cognitive outcomes. Although numerous promising biomarkers have already been identified, larger multi-center studies, such as the ongoing DISCOVERY STUDY (Rost et al., 2021) and DEMDAS (Filler et al., 2025) are essential to validate their utility in prediction, diagnosis, and potentially even therapeutic interventions, and to ensure their applicability across diverse patient populations.

## Data Availability

The original contributions presented in this study are included in the article/Supplementary material, further inquiries can be directed to the corresponding author.
