# Supplementary material for: Biomarkers of post-stroke cognitive impairment—a systematic literature review
Source: Front Behav Neurosci. 2026 Jun 22;20:1864277. doi: 10.3389/fnbeh.2026.1864277 (PMC13333719; doi:10.3389/fnbeh.2026.1864277)
Supplement: Supplementary file 1 [file Data_Sheet_1.docx]

**Supplementary material**

**Supplementary table 1: Body-fluid biomarkers**

| **Author, year** | **Ethnicity** | **Study design** | **Sample size** | **Sex (% female)** | **Age (mean)** | **Consortium name** | **Stroke etiology** | **Pattern Ischemic Stroke** | **First Stroke** | **Biomarker measurement time** | **PSCI evaluation after stroke** | **PSCI testing tool** | **Adjusted effect size for primary outcome (if applicable)** | **p-value** | **Biomarkers** |
| --- | --- | --- | --- | --- | --- | --- | --- | --- | --- | --- | --- | --- | --- | --- | --- |
| **Blood**  **Inflammation & Oxidative stress** |  |  |  |  |  |  |  |  |  |  |  |  |  |  |  |
| Cogo et al. (2021) | white,asian | 1 | 23 | 39% | 67 |  | LAA,CES,  CYP | embolic,NS | NS | At hospitalization | 3m | MMSE, MoCA, Wais-IV, FAB, TMT-B, trail B-trail A, ROCF, VST | median difference, Mann Whitney U effect size 0.88 | < 0.0001 | QUIN/KYNA ratio |
| Cogo et al. (2021) | white,asian | 1 | 23 | 39% | 67 |  | LAA,CES,  CYP | embolic,NS | NS | At hospitalization | 3m | MMSE, MoCA, Wais-IV, FAB, TMT-B, trail B-trail A, ROCF, VST | median difference, Mann Whitney U effect size 0.67 | 0.0004 | QUIN |
| Rothenburg et al. (2010) | white | 3 | 48 | 46% | 72 | 4 stroke centers | NS | NS | NS | 5-30d | 5-30d | MMSE | β-coefficient -0.46 | 0.002 | CRP |
| Kulesh et al. (2018) | white | 3 | 57 | 35% | 65 |  | LAA,CES,  SVD,CYP | embolic,  lacunar,NS | NS | 4-21d | 7-14d | MMSE, MoCA, FAB, SVF, Five Word Test, CDT, Schulte Test | NS | 0.011 (DCI compared to NC) | IL-6 |
| Chen et al. (2019) | asian | 3 | 117 | 37% | 66 |  | LAA,SVD | lacunar,NS | NS | >30d | >30d | NINDS-AIREN | NS | 0.001 | low BChE Activity |
| Zhu et al. (2019) | asian | 1 | 638 | 30% | 61 | CATIS | LAA,CES,  SVD | embolic,  lacunar,NS | yes | <72h | 3m | MMSE | OR 1.22, 95% CI 1.02–1.46 | 0.03 | RF |
| Wang et al. (2021) | asian | 3 | 69 | 12% | 63 |  | NS | NS | NS | <72h | <7d | MMSE | HR 0.945, 95% CI 0.907–0.984 | 0.006 | low LXA4 |
| Wang et al. (2021) | asian | 1 | 416 | 40% | 65 |  | LAA,CES,  SVD,CYP,  OTH | embolic,lacunar,NS | yes | <24h of admission | 3m | MoCA | OR 5.49, 95% CI 3.62–5.97 | <0.001 | Galectin-3 |
| Shan et al. (2021) | asian | 1 | 276 | 45% | 67 |  | LAA,CES,  SVD,CYP,  OTH | embolic,  lacunar,NS | yes | <14d (imaging),<8d (serum) | 3m | MMSE | OR 1.51, 95% CI 1.12–2.04 | 0.006 | WBC |
| Ye et al. (2022) | asian | 1 | 219 | 53% | 70 |  | SVD | lacunar | NS | <8d | 3m | MMSE | OR 1.031, 95% CI 1.009–1.054 | 0.007 | Amyloid A |
| Ye et al. (2022) | asian | 1 | 219 | 53% | 70 |  | SVD | lacunar | NS | <8d | 3m | MMSE | OR 1.271, 95% CI 1.028–1.572 | 0.027 | WBC |
| Shang et al. (2022) | asian | 1 | 454 | 28% | 62 |  | LAA,CES,  SVD,CYP,  OTH | embolic,  lacunar,NS | no | <24h | 7-10d | MoCA | OR 1.025, 95% CI 1.005-1.406 | <0.016 | Neutrophil percentage |
| Shang et al. (2022) | asian | 1 | 454 | 28% | 62 |  | LAA,CES,  SVD,CYP,  OTH | embolic,  lacunar,NS | no | <24h | 7-10d | MoCA | OR 2.167, 95% CI 1.127-4.166 | <0.05 | Neutrophil-lymphocyte ratio (NLR) |

| Sandvig et al. (2024) | white | 1 | 422 | 41% | 72 | Nor-COAST | LAA,CES,  SVD,CYP,  OTH | embolic,  lacunar,NS | no | At hospitalization | 18m,36m | MoCA | B-coefficient −0.163, 95% CI −0.242-(−0.083) (18m); B-coefficient −0.169, 95% CI −0.256-(−0.083) (36m) | <0.001 (18m, 36m) | Neopterin |
| --- | --- | --- | --- | --- | --- | --- | --- | --- | --- | --- | --- | --- | --- | --- | --- |
| Sandvig et al. (2024) | white | 1 | 422 | 41% | 72 | Nor-COAST | LAA,CES,  SVD,CYP,  OTH | embolic,  lacunar,NS | no | At hospitalization | 3m,18m,36m | MoCA | B-coefficient −0.005, 95% CI -0.007-(−0.002) (3m); B-coefficient −0.006, 95% CI −0.008-(−0.003) (18m); B-coefficient −0.004, 95% CI −0.007-(−0.002) (36m) | <0.001 (3m, 18m); 0.002 (36m) | QUIN |
| Sandvig et al. (2024) | white | 1 | 422 | 41% | 72 | Nor-COAST | LAA,CES,  SVD,CYP,  OTH | embolic,  lacunar,NS | no | At hospitalization | 18m,36m | MoCA | B-coefficient −0.034, 95% CI −0.054-(−0.014) (18m); B-coefficient −0.031, 95% CI −0.053-(−0.008) (36m) | 0.001 (18m); 0.009 (36m) | HK |
| Sandvig et al. (2024) | white | 1 | 422 | 41% | 72 | Nor-COAST | LAA,CES,  SVD,CYP,  OTH | embolic,  lacunar,NS | no | At hospitalization | 36m | MoCA | B-coefficient 0.095, 95% CI 0.023-0.168 (36m) | 0.009 (36m) | XA |
| Sandvig et al. (2024) | white | 1 | 422 | 41% | 72 | Nor-COAST | LAA,CES,  SVD,CYP,  OTH | embolic,  lacunar,NS | no | At hospitalization | 3m,18m,36m | MoCA | B-coefficient −0.047, 95% CI -0.074-(−0.020) (3m); B-coefficient −0.060, 95% CI −0.089-(−0.031) (18m); B-coefficient −0.065, 95% CI −0.097-(−0.034) (36m) | 0.001 (3m); <0.001 (18m, 36m) | HK-ratio |
| Sandvig et al. (2024) | white | 1 | 422 | 41% | 72 | Nor-COAST | LAA,CES,  SVD,CYP,  OTH | embolic,  lacunar,NS | no | At hospitalization | 3m,18m,36m | MoCA | B-coefficient −3.042, 95% CI -4.868-(−1.216) (3m); B-coefficient −3.857, 95% CI −5.800-(−1.915) (18m); B-coefficient −3.729, 95% CI −5.804-(−1.653) (36m) | 0.001 (3m);  <0.001 (18m, 36m) | PA-ratio |
| Sandvig et al. (2024) | white | 1 | 422 | 41% | 72 | Nor-COAST | LAA,CES,  SVD,CYP,  OTH | embolic,  lacunar,NS | no | 3m | 18m,36m | MoCA | B-coefficient -0.139, 95% CI −0.220-(−0.058) (18m); B-coefficient −0.143, 95% CI −0.230-(−0.056) (36m) | 0.001 | Neopterin |

| Sandvig et al. (2024) | white | 1 | 422 | 41% | 72 | Nor-COAST | LAA,CES,  SVD,CYP,  OTH | embolic,  lacunar,NS | no | 3m | 18m,36m | MoCA | B-coefficient 0.027, 95% CI 0.007-0.046(18m); B-coefficient 0.032, 95% CI 0.011-0.053 (36m) | 0.008 (18m); 0.003 (36m) | Pyridoxal 5́-phosphate |
| --- | --- | --- | --- | --- | --- | --- | --- | --- | --- | --- | --- | --- | --- | --- | --- |
| Sandvig et al. (2024) | white | 1 | 422 | 41% | 72 | Nor-COAST | LAA,CES,  SVD,CYP,  OTH | embolic,  lacunar,NS | no | 3m | 36m | MoCA | B-coefficient 0.035, 95% CI 0.010-0.059 (36m) | 0.005 | low Picolinic acid |
| Gold et al. (2011) | white | 3 | 41 | 46% | 72 |  | NS | NS | NS | <1m (mean 9.17d) | mean 9.17d | MMSE | β-coefficient -0.412 | 0.006 | K/T-ratio |
| Cheng et al. (2024) | asian | 1 | 332 | 39% | 68 |  | LAA,CES,  SVD,CYP,  OTH | embolic,  lacunar,NS | yes | <8d | 3m | MoCA | OR 10.369, 95% CI 4.460–24.107 | <0.001 | systemic immune-inflammation index (SII) |
| Liu et al. (2017) | asian | 1 | 193 | 38% | median |  | LAA,CES,  SVD,CYP | embolic,  lacunar,NS | yes | <8d | 1m | MMSE | OR 6.261, 95%CI 2.766–14.176 | <0.001 | 8-OHdG |
| Liu et al. (2017) | asian | 1 | 193 | 38% | median |  | LAA,CES,  SVD,CYP | embolic,  lacunar,NS | yes | <8d | 1m | MMSE | OR 14.130, 95% CI 5.250-38.032 | <0.001 | MDA |
| [Chen et al. (2019)](https://pubmed.ncbi.nlm.nih.gov/?term=Chen+YC&cauthor_id=31105635) | asian | 3 | 73 | 29% | 64 |  | LAA,SVD | embolic,  lacunar | NS | >30d | >30d | NINDS-AIREN, MMSE | β-coefficient 86.19, SE 26.60 | 0.002 | DAO |
| Liu et al. (2019) | asian | 1 | 134 | 34% | 61 |  | LAA,CES,  SVD,OTH | embolic,  lacunar,NS | no | <7d | 3m | MMSE | OR 2.000, 95% CI 1.062–3.770 | 0.032 | FIB |
| Qian et al. (2012) | asian | 2 | 152 | 43% | 67 |  | NS | NS | yes | 6-72h | 2w | MMSE, MoCA | OR 0.992, 95% CI 0.990–0.995 | 0.001 | low sRAGE |
| Zhu et al. (2019) | asian | 1 | 638 | 30% | 61 | CATIS | LAA,CES,  SVD | embolic,  lacunar,NS | yes | <72h | 3m | MMSE | OR 1.47, 95% CI 1.22–1.77 | <0.001 | MMP-9 |
| Ge et al. (2020) | asian | 1 | 598 | 31% | 60 | seven hospitals from CATIS | LAA,CES,  SVD | embolic,  lacunar,NS | yes | <3d | 3m | MMSE | OR 1.80, 95% CI 1.09-2.97 | <0.01 | TIMP-1 |
| Ge et al. (2020) | asian | 1 | 598 | 31% | 60 | seven hospitals from CATIS | LAA,CES,  SVD | embolic,  lacunar,NS | yes | <3d | 3m | MoCA | OR 2.55, 95% CI 1.49-4.35 | <0.001 | TIMP-1 |
| Zhao et al. (2022) | asian | 2 | 467 | 65% | NS |  | LAA,CES,  SVD,CYP,  OTH | embolic,  lacunar,NS | NS | 3m-6m | 3m | MoCA | OR 1.01, 95% CI 1.01–1.02 | <0.001 | MMP-9 |
|  |  |  |  |  |  |  |  |  |  |  |  |  |  |  |  |
| **Blood glucose (diabetes associated)** |  |  |  |  |  |  |  |  |  |  |  |  |  |  |  |
| Shang et al. (2020) | white | 1 | 381 | NS | ≥60 | SNAC-K | HS,NS | NS | NS | NS | y to dementia (every 3y or 6y, up to 12y) | DSM-IV | HR 2.56, 95% CI 1.04-6.25 | NS | Diabetes mellitus |
| Yang et al. (2014) | asian | 1 | 1013 | 44% | 69 |  | LAA,CES,  SVD,OTH,  HS**,(TIA)** | embolic,  lacunar,NS | no | <1w of admission | 3-6m | MoCA, MMSE | OR 1.904, 95% CI 1.147-3.160 | 0.013 | Diabetes mellitus |

| Wang et al. (2017) | asian | 1 | 201 | 35% | median |  | LAA,CES,  SVD,CYP,  OTH | embolic,  lacunar,NS | no | <8d | 1m | MMSE | OR 3.062, 95% CI 1.130-8.299 | 0.028 | Prediabetes |
| --- | --- | --- | --- | --- | --- | --- | --- | --- | --- | --- | --- | --- | --- | --- | --- |
| Wang et al. (2017) | asian | 1 | 201 | 35% | median |  | LAA,CES,  SVD,CYP,  OTH | embolic,  lacunar,NS | no | <8d | 1m | MMSE | OR 4.798, 95% CI 1.360-16.925 | 0.015 | Diabetes mellitus |
| Lim et al. (2018) | asian | 1 | 354 | 35% | 63 |  | LAA,CES,  SVD,CYP,  OTH | embolic,  lacunar,NS | NS | <7d | 3m | K-VCIHS-NP | OR 1.94, 95% CI 1.11–3.42 | <0.05 | MAG in diabetes group |
| Ding et al. (2019) | asian | 1 | 145 | 29% | median |  | LAA,CES,  SVD,CYP,  OTH | embolic,  territorial,  lacunar | no | <7d | 6-12m | MMSE, MoCA, or a neuropsychological battery | OR 5.825, 95% CI 2.068-16.412 | 0.001 | Diabetes mellitus |
| Yuan et al. (2021) | asian | 1 | 376 | 52% | 66 | 3 hospitals | NS | NS | no | once stabilized after stroke | At admission,1m,2m,3m,4m,5m,6m,7m,8m,9m,10m,11m,12m | MoCA | OR 4.13, 95% CI 2.63–7.54 | NS | History of diabetes |
| Zhao et al. (2022) | asian | 2 | 467 | 65% | NS |  | LAA,CES,  SVD,CYP,  OTH | embolic,  lacunar,NS | NS | 3m-6m | 3m | MoCA | OR 1.77, 95% CI 1.07–2.93 | 0.03 | Diabetes mellitus |
| Ma et al. (2022) | asian | 3 | 161 | 46% | 68 |  | LAA,CES,  SVD,CYP,  OTH | embolic,  territorial,  lacunar | no | <72h | <72h | MoCA | OR 5.39, 95% CI 2.40-14.59 | <0.001 | Diabetes duration |
| Ma et al. (2022) | asian | 3 | 161 | 46% | 68 |  | LAA,CES,  SVD,CYP,  OTH | embolic,  territorial,  lacunar | no | <72h | <72h | MoCA | OR 3.89, 95% CI 1.66-10.04 | 0.003 | HbA1c |
| Ohlmeier et al. (2022) | white | 2 | 1337 | 41% | 65 | DNA Lacunar 2 Study | SVD | lacunar | no | <1y (median 2d) | median 37d | BMET | OR 1.98, 95% CI 1.40–2.80 | 0.001 | Diabetes mellitus |
| Ye et al. (2022) | asian | 1 | 219 | 53% | 70 |  | SVD | lacunar | NS | <8d | 3m | MMSE | OR 2.679, 95% CI 1.029–6.976 | 0.044 | Diabetes mellitus |
| Kang et al. (2023) | asian | 1 | 37 | 32% | 72 |  | NS | NS | yes | 3m | 3m,12m | K-VCIHS-NP, MMSE | OR 32.9, 95% CI 2.15–2962.9 | 0.042 | Diabetes mellitus |
| Xu et al. (2023) | asian | 1 | 311 | 36% | 67 |  | NS | NS | yes | <8d | 3m-6m | MoCA | OR 1.96, 95% CI 1.08-3.58 | 0.028 | HbA1c >8.2% |
| Zhou et al. (2024) | asian | 1 | 554 | 31% | 62 |  | NS | NS | no | morning after <24h,every d (average 5.52d,range 2-15d) | 7d | MoCA | OR 1.048, 95% CI 1.208−1.818 | 0.047 | HbA1c |
| Zhou et al. (2024) | asian | 1 | 554 | 31% | 62 |  | NS | NS | no | morning after <24h,every d (average 5.52d,range 2-15d) | 7d | MoCA | OR 53.692, 95% CI 10.680−269.302 | <0.001 | SHR |
| Zhou et al. (2024) | asian | 1 | 554 | 31% | 62 |  | NS | NS | no | morning after <24h,every d (average 5.52d,range 2-15d) | 7d | MoCA | OR 0.628, 95% CI 0.372−0.975 | 0.039 | low TIR |

| Zhou et al. (2024) | asian | 1 | 554 | 31% | 62 |  | NS | NS | no | morning after <24h,every d (average 5.52d,range 2-15d) | 7d | MoCA | OR 1.658, 95% CI 1.388−1.982 | <0.001 | MPBG |
| --- | --- | --- | --- | --- | --- | --- | --- | --- | --- | --- | --- | --- | --- | --- | --- |
| Zhou et al. (2024) | asian | 1 | 554 | 31% | 62 |  | NS | NS | no | morning after <24h,every d (average 5.52d,range 2-15d) | 7d | MoCA | OR 2.697, 95% CI 2.047−3.555 | <0.001 | MAGE |
| **Metabolic** |  |  |  |  |  |  |  |  |  |  |  |  |  |  |  |
| Wu et al. (2020) | asian | 1 | 487 | 51% | 72 |  | NS | NS | no | At admission | 3y | DSM-IV | OR 0.83, 95% CI 0.6-1.148 | 0.051 | LDL |
| Wu et al. (2020) | asian | 1 | 487 | 51% | 72 |  | NS | NS | no | At admission | 3y | DSM-IV | OR 1.018, 95% CI 0.944-1.042 | 0.01 | Hcy |
| Wu et al. (2020) | asian | 1 | 487 | 51% | 72 |  | NS | NS | no | At admission | 3y | DSM-IV | OR 1.00, 95% CI 0.998-1.002 | 0.007 | UA |
| Jiang et al. (2014) | asian | 2 | 162 | 38% | 63 |  | HS,NS | NS | NS | <48h | 7d,1m,3m | MoCA | r = −0.468 | 0.038 | tHcy |
| Jiang et al. (2014) | asian | 2 | 162 | 38% | 63 |  | HS,NS | NS | NS | <48h | 7d,1m,3m | MoCA | r = 0.509 | 0.022 | low Folate |
| Jiang et al. (2014) | asian | 2 | 162 | 38% | 63 |  | HS,NS | NS | NS | <48h | 7d,1m,3m | MoCA | r = 0.588 | 0.006 | low Vitamin B12 |
| Pascoe, Linden (2016) | white | 3 | 149 | 65% | 81 |  | HS,NS | NS | NS | 20m | 20m | MMSE | β-coefficient 0.2 | <0.05 | low Folate |
| Pascoe, Linden (2016) | white | 3 | 149 | 65% | 81 |  | HS,NS | NS | NS | 20m | 20m | MMSE | β-coefficient -0.22 | <0.05 | MMA |
| Chen et al. (2018) | asian | 1 | 314 | 36% | 63 |  | LAA,CES,  SVD,CYP | embolic,  lacunar,NS | yes | <8d | 1m | MMSE | OR 4.319, 95% CI 1.553-12.013 | 0.005 | low T3 (syndrome) |
| He et al. (2020) | asian | 1 | 326 | 36% | 65 |  | LAA,CES,  SVD,CYP | embolic,  lacunar,NS | no | <8d | 1m | MMSE | OR 3.637, 95% CI 1.216-10.881 | 0.021 | Anemia (Hb) |
| Yan et al. (2017) | asian | 4 | 104 | 46% | 53 |  | NS | NS | no | <24h | 1m | MMSE | OR 3.563, 95%CI 0.799–5.637 | 0.036 | Hcy |
| Zhu et al. (2019) | asian | 1 | 264 | 46% | 67 |  | LAA,CES,  SVD,OTH | embolic,  lacunar,NS | yes | <8d | 1y | MMSE | OR 3.304, 95% CI 1.335–8.178 | 0.01 | TMAO |
| Lu et al. (2019) | asian | 1 | 213 | 35% | 65 |  | LAA,CES,  SVD,OTH | embolic,  lacunar,NS | yes | <8d | 3m | MoCA | OR 1.063, 95% CI 1.109–1.109 | 0.005 | Hcy |
| Lu et al. (2019) | asian | 1 | 213 | 35% | 65 |  | LAA,CES,  SVD,OTH | embolic,  lacunar,NS | yes | <8d | 3m | MoCA | OR 7.797, 95% CI 2.917–20.843 | 0 | hypertension with Hcy |
| von Rennenberg et al. (2024) | white | 1 | 385 | 32% | 68 (median) | DEMDAS | LAA,CES,  SVD,CYP,  OTH | embolic,  lacunar,NS | NS | <5d | 12m | TMT-A,DSST | β-coefficient 0.273, 95% CI -0.436-(-0.109) (for attention) | <0.05 | hs-cTnT |
| Hou et al. (2019) | asian | 1 | 261 | 46% | 66 | 3 hospitals | LAA,CES,  SVD,OTH | embolic,  lacunar,NS | yes | <8d | 3m | MoCA | OR 1.97, 95% CI 1.01-3.83 | 0.046 | low RA |
| Zhu et al. (2019) | asian | 1 | 638 | 30% | 61 | CATIS | LAA,CES,  SVD | embolic,  lacunar,NS | yes | <72h | 3m | MMSE | OR 1.22, 95% CI 1.01–1.49 | 0.044 | tHcy |
| Ling et al. (2020) | asian | 1 | 93 | 33% | 70 |  | NS | NS | no | <1w of admission | 3m | MoCA | OR 1.219, 95% CI 1.013–1.466 | 0.036 | Hcy |
| Feng et al. (2020) | asian | 3 | 182 | 38% | 63 |  | LAA,CES,  SVD,OTH | embolic,  lacunar,NS | no | <8d | <7d | MMSE | OR 2.033, 95% CI 1.017–4.067 | 0.045 | low Thiamine (Vitamin B1) |
| Sun et al. (2020) | asian | 3 | 274 | 38% | 69 |  | NS | NS | NS | <7d | 1w | MoCA | B-coefficient 0.007, SE 0.002, OR 1.007 | 0.005 | UA |
| Mao et al. (2020) | asian | 1 | 195 | 44% | 69 |  | LAA,CES,  SVD,CYP,  OTH | embolic,  lacunar,NS | no | <8d | 1y | MoCA | RR 0.430, 95% CI 0.332-0.918 | 0.038 | Low Triiodothyronine (T3) |
| Zhao et al. (2022) | asian | 2 | 467 | 65% | NS |  | LAA,CES,  SVD,CYP,  OTH | embolic,  lacunar,NS | NS | 3m-6m | 3m | MoCA | OR 1.88, 95% CI 1.18–3.56 | 0.01 | Hcy |
| Ma et al. (2022) | asian | 3 | 161 | 46% | 68 |  | LAA,CES,  SVD,CYP,  OTH | embolic,  territorial,  lacunar | no | <72h | <72h | MoCA | OR 0.20, 95% CI 0.08-0.42 | <0.001 | low Albumin |
| Yang et al. (2022) | white | 1 | 17729 | 45% | 72 | Clinical Practice Research Datalink (CPRD) | HS,NS | NS | yes | 3m before - 3m after index stroke | median 4.6y | ICD-10 | HR 1.29, 95% CI 1.14–1.47 | <0.001 | LDL-C |
| Yang et al. (2022) | white | 1 | 17729 | 45% | 72 | Clinical Practice Research Datalink (CPRD) | HS,NS | NS | yes | 3m before - 3m after index stroke | median 4.6y | ICD-10 | HR 0.79, 95% CI 0.69–0.89 | <0.001 | low TG |
| Xu et al. (2022) | asian | 3 | 313 | 33% | 66 (median) |  | NS | NS | no | <24h | <2w | MMSE | OR 0.297, 95% CI 0.136-0.649 | 0.002 | low Iron |
| Cheng et al. (2022) | asian | 1 | 227 | 41% | 66 |  | LAA,CES,  SVD,CYP | embolic,  lacunar,NS | yes | <24h of admission | 3m | MoCA | OR 3.940, 95% CI 2.337–6.664 | <0.001 | TG/HDL-C Ratio |
| Zhao et al. (2022) | asian | 2 | 180 | 43% | 68 |  | HS,NS | NS | yes | 3-12m | 3-12m | MoCA, NS | OR 5.026, 95% CI 1.198–21.089 | 0.027 | Hcy |
| Lee et al. (2023) | asian | 3 | 264 | 36% | 66 |  | LAA | embolic,NS | no | <8d | 3m-6m | K-VCIHS-NP | OR 3.16, 95% CI 1.19-8.41 | 0.021 | ApoB/ApoA-I ratio |
| Cheng et al. (2023) | asian | 1 | 313 | 38% | 68 |  | LAA,CES,  SVD,CYP | embolic,NS | yes | morning after <72h | 3m | MoCA | OR 5.648, 95% CI 3.247-9.824 | <0.001 | Triglyceride-glucose index (TyG) |
| Cheng et al. (2023) | asian | 1 | 313 | 38% | 68 |  | LAA,CES,  SVD,CYP | embolic,NS | yes | morning after <72h | 3m | MoCA | OR 0.299, 95% CI 0.114-0.781 | 0.014 | HDL-C |
| Gallucci et al. (2024) | white | 1 | 329 | 41% | 67 |  | NS | territorial | yes | At hospitalization | <10d | Learning of 10 words, Copy of four figures, Ten words recall and recognition, Four figures recall, DGS, TMT-A/B, Initial letter fluency “S”, Computerized alertness test, Bells test, total number of omissions, Center of Cancellation | OR 1.93, 95% CI 1.01–3.68 | 0.046 | Hyperlipidemia |
| Gu et al. (2022) | asian | 1 | 123 | 38% | 67 |  | HS |  | NS | At admission | 6m | MoCA | OR 0.166, 95% CI 0.038–0.725 | 0.017 | low Hemoglobin |
|  |  |  |  |  |  |  |  |  |  |  |  |  |  |  |  |

| **Protein biomarkers** |  |  |  |  |  |  |  |  |  |  |  |  |  |  |  |
| --- | --- | --- | --- | --- | --- | --- | --- | --- | --- | --- | --- | --- | --- | --- | --- |
| Qian et al. (2012) | asian | 2 | 152 | 43% | 67 |  | NS | NS | yes | 6-72h | 2w | MMSE, MoCA | OR 1.007, 95% CI 1.002–1.013 | 0.005 | BACE1 |
| Tang et al. (2018) | asian | 2 | 61 | 48% | 74 |  | NS | NS | NS | mean 84.17m | 71.2 ± 70.3m in stroke patients without dementia,100.5 ± 61.0m in stroke patients with dementia | NINDS-AIREN | OR 1.85, 95% CI 1.25–2.75 | 0.002 | Aβ-42 |
| Mao et al. (2020) | asian | 1 | 195 | 44% | 69 |  | LAA,CES,  SVD,CYP,  OTH | embolic,  lacunar,NS | no | <8d | 1y | MoCA | RR 0.282, 95% CI 0.258-0.833 | 0.012 | Low Aβ 1-42 |
| Qian et al. (2020) | asian | 1 | 613 | 30% | 60 | CATIS | LAA,CES,  SVD | embolic,  lacunar,NS | yes | <72h | 3m | MoCA | OR 2.01, 95% CI 1.15–3.53 | 0.013 | Endostatin |
| Prodjohardjono et al. (2020) | asian | 1 | 56 | 38% | 61 |  | NS | embolic,NS | yes | 5d | 3m | MoCA | OR 4.99, 95% CI 1.01–24.7 | 0.048 | VEGF |
| Jia et al. (2020) | asian | 1 | 1019 | 48% | 64 |  | LAA,CES,  SVD,CYP | embolic,  lacunar,NS | yes | <8d | 3m | MMSE | OR 1.42, 95% CI 1.17–3.09 | 0.012 | ALP |
| Chen et al. (2021) | asian | 1 | 193 | 36% | 68 |  | LAA,CES,  SVD,CYP,  OTH | embolic,  lacunar,NS | yes | <3d | 3m | MMSE | OR 1.001, 95% CI 1.000–1.003 | 0.043 | LMP2 |
| [Huang et al. (2022)](https://pubmed.ncbi.nlm.nih.gov/?term=Huang+LK&cauthor_id=35572134) | asian | 1 | 136 | 29% | 59 |  | LAA,CES,  SVD,CYP,  OTH | embolic,  lacunar,NS | yes | <7d | 3m | MoCA | OR 0.62, 95% CI 0.40–0.94 | 0.0243 | low p-tau181 |
| [Huang et al. (2022)](https://pubmed.ncbi.nlm.nih.gov/?term=Huang+LK&cauthor_id=35572134) | asian | 1 | 136 | 29% | 59 |  | LAA,CES,  SVD,CYP,  OTH | embolic,  lacunar,NS | yes | <7d | 12m | MoCA | OR 0.69, 95% CI 0.47–0.99 | 0.0443 | low p-tau181 |
| Gao et al. (2022) | asian | 1 | 316 | 38% | 66 |  | HS |  | NS | <24h | 3m | MoCA | β-coefficient 0.311 | 0.038 | low Neuroglobin |
| Sadhukhan et al. (2023) | indian | 2 | 411 | 18% | 56 | 2 hospitals | NS | NS | no | NS | 6-12m | MMSE | NS | 0.0337 | low mBDNF |
| Chang et al. (2024) | asian | 1 | 593 | 31% | 60 | CATIS | LAA,CES,  SVD | embolic,  lacunar | NS | <72h | 3m | MMSE | OR 0.60, 95% CI 0.39–0.94 | 0.024 | low BDNF |
| You et al. (2022) | asian | 1 | 600 | 30% | 60 | CATIS | LAA,SVD | embolic,  lacunar | yes | <72h | 3m | MoCA | OR 0.49, 95% CI 0.29–0.81 | 0.011 | low sDPP4 |
|  |  |  |  |  |  |  |  |  |  |  |  |  |  |  |  |
| **Cellular** |  |  |  |  |  |  |  |  |  |  |  |  |  |  |  |
| Kirkpatrick et al. (2019) | white,black | 1 | 128 | 8% | 66 |  | LAA,CES,  CYP,OTH | embolic,NS | no | <24h | 3m | MMSE | r = -0.078, 95% CI -0.12- (-0.041) | <0.0001 | 10% higher platelet count |
| Gong et al. (2020) | asian | 1 | 141 | 36% | 67 |  | HS |  | NS | <2w | 12-14d | MoCA | OR 1.079, 95% CI 1.002-1.162 | 0.043 | MCV |
| Gu et al. (2022) | asian | 1 | 123 | 38% | 67 |  | HS |  | NS | At admission | 6m | MoCA | OR 10.204, 95% CI 2.369–43.95 | 0.002 | MCV |
| Gu et al. (2022) | asian | 1 | 123 | 38% | 67 |  | HS |  | NS | At admission | 6m | MoCA | OR 18.055, 95% CI 3.784–86.151 | <0.001 | RDW |
|  |  |  |  |  |  |  |  |  |  |  |  |  |  |  |  |
| **Damage Marker** |  |  |  |  |  |  |  |  |  |  |  |  |  |  |  |
| Wang et al. (2021) | asian | 1 | 1694 | 47% | 64 |  | LAA,CES,  SVD,CYP,  OTH | embolic,  lacunar,NS | yes | <48h | 3m | MoCA | OR 1.041, 95% CI 1.034–1.047 | <0.001 | pNfL |
| Jiang et al. (2022) | asian | 1 | 264 | 41% | 65 (median) |  | LAA,CES,  SVD,CYP,  OTH | embolic,  lacunar,NS | yes | <48h | 90d | MoCA | OR 1.035, 95% CI 1.019–1.051 | <0.001 | pNfL |
| Liu et al. (2024) | asian | 1 | 192 | 31% | 67 |  | NS | NS | yes | <7d | 3m | MoCA | β-coefficient 0.236 | <0.001 | HMGB1 |
| Qu et al. (2024) | asian | 1 | 180 | 37% | 68 |  | NS | NS | no | At admission | 1y,2y,3y | MMSE | Z score= -2.083 | 0.037 | ATG-5 |
| Yan et al. (2022) | asian | 1 | 281 | 31% | 60 |  | LAA,CES,  SVD,CYP,  OTH | embolic,  lacunar,NS | yes | 8-24h | 3m | MoCA | OR 5.745, 95% CI 1.089-30.311 | 0.039 | CysC |
| Ye et al. (2022) | asian | 1 | 219 | 53% | 70 |  | SVD | lacunar | NS | <8d | 3m | MMSE | OR 3.118, 95% CI 1.053–9.228 | 0.04 | CysC |
|  |  |  |  |  |  |  |  |  |  |  |  |  |  |  |  |
| **Combination Inflammation, Metabolic** |  |  |  |  |  |  |  |  |  |  |  |  |  |  |  |
| Zhu et al. (2019) | asian | 1 | 638 | 30% | 61 | CATIS | LAA,CES,  SVD | embolic,  lacunar,NS | yes | <72h | 3m | MMSE | OR 4.89, 95% CI 2.31–10.35 | <0.001 | RF, MMP-9, tHcy |
|  |  |  |  |  |  |  |  |  |  |  |  |  |  |  |  |
|  |  |  |  |  |  |  |  |  |  |  |  |  |  |  |  |
| **CSF** |  |  |  |  |  |  |  |  |  |  |  |  |  |  |  |
| **Inflammation & Oxidative stress** |  |  |  |  |  |  |  |  |  |  |  |  |  |  |  |
| Kulesh et al. (2018) | white | 3 | 57 | 35% | 65 |  | LAA,CES,  SVD,CYP | embolic,  lacunar,NS | NS | 4-21d | 7-14d | MMSE, MoCA, FAB, SVF, Five Word Test, CDT, Schulte Test | NS | 0.033 (DCI compared to NC) | IL-1β |
| Kulesh et al. (2018) | white | 3 | 57 | 35% | 65 |  | LAA,CES,  SVD,CYP | embolic,  lacunar,NS | NS | 4-21d | 7-14d | MMSE, MoCA, FAB, SVF, Five Word Test, CDT, Schulte Test | NS | 0.05 (DCI compared to NC) | IL-10 |
|  |  |  |  |  |  |  |  |  |  |  |  |  |  |  |  |
|  |  |  |  |  |  |  |  |  |  |  |  |  |  |  |  |
| **Urine** |  |  |  |  |  |  |  |  |  |  |  |  |  |  |  |
| **Metabolic** |  |  |  |  |  |  |  |  |  |  |  |  |  |  |  |
| Tong et al. (2017) | asian | 2 | 357 | NS | 76 | two rural and urban districts, a geriatric hospital for dementia, and 5 homes for the elderly in Beijing, China | HS,NS | NS | NS | 3m-2y | 3m-2y | MMSE, (DSM-IV-R) | NS | 0 | urine formaldehyd concentration (cut-off concentration 0.0418 mM) |
| **Saliva** |  |  |  |  |  |  |  |  |  |  |  |  |  |  |  |
| **Inflammation & Oxidative stress** |  |  |  |  |  |  |  |  |  |  |  |  |  |  |  |
| Zhanina et al. (2022) | white | 1 | 45 | 27% | 56 |  | NS | NS | yes | <48h | <48h,30d,  180d,365d (control group only at admission) | MoCA, ST, LMWT, Head’s probe, ROCF | OR 1.42, 95% CI 1.11–1.99 | 0.0185 | Cortisol |
|  |  |  |  |  |  |  |  |  |  |  |  |  |  |  |  |
|  |  |  |  |  |  |  |  |  |  |  |  |  |  |  |  |
| **Stool** |  |  |  |  |  |  |  |  |  |  |  |  |  |  |  |
| **Inflammation & Oxidative stress** |  |  |  |  |  |  |  |  |  |  |  |  |  |  |  |
| Wang et al. (2022) | asian | 1 | 83 | 18% | 57 |  | LAA,NS,HS | embolic,NS | no | <4d,3m | 3m | MoCA | OR 1.476, 95% CI 1.089-1.690 | 0.006 | Abundance of Enterobacteri-aceae |

**Supplementary table 2: Genetic biomarkers**

| **Author, year** | **Ethnicity** | **Study design** | **Sample size** | **Sex (% female)** | **Age (mean)** | **Consortium name** | **Stroke etiology** | | **Pattern Ischemic Stroke** | | **First Stroke** | | **Biomarker measurement time** | | **PSCI evaluation after stroke** | | **PSCI testing tool** | **Adjusted effect size for primary outcome (if applicable)** | **p-value** | **Biomarkers** |
| --- | --- | --- | --- | --- | --- | --- | --- | --- | --- | --- | --- | --- | --- | --- | --- | --- | --- | --- | --- | --- |
| **DNA/RNA**  **SNP** |  |  |  |  |  |  |  | |  | |  | |  | |  | |  |  |  |  |
| Zhao et al. (2022) | asian | 2 | 467 | 65% | NS |  | LAA,CES,  SVD,CYP,  OTH | | embolic,  lacunar,NS | | NS | | 3m-6m | | 3m | | MoCA | OR 3.91, 95% CI 1.63–9.38 | 0.002 | TC genotype carrying MMP-9 rs3918242 |
| Zhao et al. (2022) | asian | 2 | 467 | 65% | NS |  | LAA,CES,  SVD,CYP,  OTH | | embolic,  lacunar,NS | | NS | | 3m-6m | | 3m | | MoCA | OR 2.79, 95% CI 1.12–7.00 | 0.028 | CC genotype carrying MMP-9 rs3918242 |
| Sadhukhan et al. (2023) | indian | 2 | 411 | 18% | 56 | 2 hospitals | NS | | NS | | no | | NS | | 6-12m | | MMSE | OR 2.502, 95% CI 1.498–4.179 | 0.001 | ‘C’ allele of rs4580704 (CLOCK gene) |
| Sadhukhan et al. (2023) | indian | 2 | 411 | 18% | 56 | 2 hospitals | NS | | NS | | no | | NS | | 6-12m | | MMSE | OR 3.226, 95% CI 1.627–6.398 | 0.001 | genotypes with C allele of rs4580704 (CLOCK gene) |
| Baum et al. (2007) | asian | 2 | 121 | 49% | 70 |  | NS | | NS | | NS | | NS | | 3m | | MMSE | OR 2.8, 95% CI 1.2-6.3 | 0.006 | GCLM –588T allele frequency |
| Klimkowicz et al. (2005) | white | 1 | 114 | 45% | 66 |  | NS | | NS | | NS | | <2d | | 3m | | DSM-IV | OR 5.06, 95% CI 1.41–18.12 | 0.013 | ACT-TT genotype |
| Klimkowicz et al. (2005) | white | 1 | 114 | 45% | 66 |  | NS | | NS | | NS | | <2d | | 3m | | DSM-IV | OR 2.68, 95% CI 1.10–6.53 | 0.03 | ACT T-Allele |
|  |  |  |  |  |  |  |  | |  | |  | |  | |  | |  |  |  |  |
| **Hereditary** |  |  |  |  |  |  |  | |  | |  | |  | |  | |  |  |  |  |
| Zhao et al. (2022) | asian | 2 | 180 | 43% | 68 |  | HS,NS | | NS | | yes | | 3-12m | | 3-12m | | MoCA, NS | OR 6.150, 95% CI 1.164–32.482 | 0.032 | APOE-ε4 allele present |
| Ma et al. (2024) | asian | 3 | 150 | 44% | 65 |  | NS | | NS | | NS | | NS | | NS | | MoCA, MMSE | OR 5.628, 95% CI 1.961-16.151 | 0.001 | APOE-ε4 allele present |
| Wagle et al. (2009) | white | 3 | 152 | 47% | 77 |  | HS,NS | | embolic,  territorial,  lacunar | | no | | mean 18.3d | | mean 18.3d | | RBANS total index score | OR 3.7, 95% CI 1.2–11.6 | 0.007 | ApoE e4 allele present |
|  |  |  |  |  |  |  |  | |  | |  | |  | |  | |  |  |  |  |
| **miRNA** |  |  |  |  |  |  |  |  | |  | |  | |  | |  | |  |  |  |
| Huang et al. (2016) | asian | 2 | 76 | 47% | 66 |  | HS,NS | NS | | yes | | <3d | | <1y | | MoCA | | r = −0.859 | < 0.001 | Serum miR-132 |
| Wang et al. (2020) | asian | 2 | 74 | 58% | 67 |  | HS,NS | NS | | yes | | <1y | | <1y | | MoCA | | r = -0.643 | <0.001 | miR-let-7i |
| Yuan et al. (2022) | asian | 3 | 77 | 47% | 66 |  | HS,NS | NS | | yes | | 14d | | 14d | | MMSE | | r = 0.752 | <0.001 | miR-21 |
| Yuan et al. (2022) | asian | 3 | 77 | 47% | 66 |  | HS,NS | NS | | yes | | 14d | | 14d | | MMSE | | r = 0.319 | <0.01 | miR-132 |
| Yuan et al. (2022) | asian | 3 | 77 | 47% | 66 |  | HS,NS | NS | | yes | | 14d | | 14d | | MMSE | | r = 0.379 | <0.01 | miR-200b |
| Wang et al. (2023) | asian | 1 | 138 | 45% | 56 |  | HS,NS | NS | | yes | | NS | | At admission,6m | | MoCA | | OR 2.804, 95% CI 1.794–5.228 | 0.005 | miR-511-3p |

**Supplementary table 3: Imaging biomarkers**

| **Author, year** | **Ethnicity** | **Study design** | **Sample size** | **Sex (% female)** | **Age (mean)** | **Consortium name** | **Stroke etiology** | **Pattern Ischemic Stroke** | **First Stroke** | **Biomarker measurement time** | **PSCI evaluation after stroke** | **PSCI testing tool** | **Adjusted effect size for primary outcome (if applicable)** | **p-value** | **Biomarkers** |
| --- | --- | --- | --- | --- | --- | --- | --- | --- | --- | --- | --- | --- | --- | --- | --- |
| **Infarct location / laterality** |  |  |  |  |  |  |  |  |  |  |  |  |  |  |  |
| Weaver et al. (2021) | white,asian | 1 | 2950 | 39% | 67 | Meta VCI Map | NS | NS | no | median 4d | <1y | multidomain neuropsychological assessment (9 cohorts) or MoCA (3 cohorts) | OR 22.857, 95% CI 2.911–179.498 | 0.003 | Infarcts in left frontotemporal lobes, left thalamus, and right parietal lobe |
| Firbank et al. (2011) | white | 1 | 106 | 54% | 80 | stroke registers from seven hospitals | NS | embolic,  territorial,  lacunar | no | 3m | y to dementia | DSM-IIIR | HR 4.11, 95% CI 1.07-15.8 | 0.04 | bilateral thalamic lesions |
| Chaudhari et al. (2014) | indian | 1 | 102 | 26% | 59 |  | LAA,CES,  CYP,HS | embolic,  lacunar,NS | no | At admission | 6m | Canadian Study of Health and Aging (VCI-ND), NINDS–AIREN (VaD) | OR 22.857, 95% CI 2.911–179.498 | 0.003 | strategic site (thalamus, angular gyrus, caudate, globus pallidus, basal forebrain, cingulate gyrus, genu or anterior limb of internal capsule or hippocampus) |
| Zhang et al. (2022) | asian | 1 | 865 | 26% | 60 | ICONS | LAA,CES,  SVD,CYP,  OTH | embolic,  lacunar,NS | yes | <1w | 12m | MoCA | OR 4.873, 95% CI 1.634-14.534 | 0.005 | Thalamic infarction |
| Cheng et al. (2023) | asian | 1 | 313 | 38% | 68 |  | LAA,CES,  SVD,CYP | embolic,NS | yes | morning after <72h | 3m | MoCA | OR 2.609, 95% CI 1.392-4.890 | 0.003 | cortical infarction |
| Ji et al. (2023) | asian | 3 | 300 | 34% | ≤45 |  | HS,NS | lacunar,NS | yes | NS | <1w | MMSE | OR 6.13, 95% CI 1.24-30.43 | <0.05 | basal ganglia infarct |
| Ji et al. (2023) | asian | 3 | 300 | 34% | ≤45 |  | HS,NS | lacunar,NS | yes | NS | <1w | MMSE | OR 7.26, 95% CI 1.58-33.40 | <0.05 | frontal temporal infarct |
| Ma et al. (2023) | asian | 1 | 120 | 45% | 63 |  | NS | NS | no | <14d | <14d | MoCA | OR 8.117, 95% CI 2.343–28.124 | 0.001 | temporal lobe infarction |
| Kandiah et al. (2011) | asian (singapore) | 1 | 145 | 23% | 56 |  | SVD | lacunar | NS | At admission | 3m | DSM IV-TR, (MoCA, MMSE, FAB) | OR 1.51, 95% CI 1.05–2.18 | 0.004 | frontal subcortical infarcts |
| Rasquin et al. (2004) | white | 1 | 176 | 43% | 68 |  | NS | territorial,  lacunar | yes | median 1d | 1m | NS | OR 2.4, 95% CI 1.0-5.7 | 0.05 | territorial infarct |
| Zhao et al. (2017) | asian | 1 | 410 | 40% | 69 |  | LAA,CES,  SVD,OTH | embolic,  lacunar,NS | no | <1w of admission | 3-6m | MoCA | β-coefficient range per mL increase: 0.0005-0.0016 | <0.0002-0.0088 | strategic brain lesion (e.g. the anterior limb of the left internal capsule and the basal ganglia) |
| Wu et al. (2023) | asian | 2 | 85 | 32% | 57 |  | NS | NS | yes | <3d | 1y | MoCA | OR 0.17, 95% CI 0.06–0.48 | <0.05 | strategic brain region infarct (thalamus, basal ganglia, frontotemporal lobes) |
| Gong et al. (2020) | asian | 1 | 141 | 36% | 67 |  | HS |  | NS | <2w | 12-14d | MoCA | OR 8.845, 95% CI 3.347-23.371 | 0.001 | dominant-hemisphere hemorrhage |
| Schellhorn et al. (2021) | white | 1 | 231 | 44% | 72 | Nor-COAST | LAA,CES,  SVD,CYP,  OTH,HS | embolic,  lacunar,NS | no | <1w | 3m | DSM-V | OR 1.8, 95% CI 1.05-3.09 | 0.032 | left hemispheric stroke |
| Gu et al. (2022) | asian | 1 | 123 | 38% | 67 |  | HS |  | NS | At admission | 6m | MoCA | OR 10.296, 95% CI 2.392–44.330 | 0.002 | Dominant-Hemisphere Hemorrhage |
| Gallucci et al. (2024) | white | 1 | 329 | 41% | 67 |  | NS | territorial | yes | At hospitalization | <10d | Learning of 10 words, copy of four figures, ten words recall and recognition, Four figures recall, DGS, TMT-A/B, Initial letter fluency “S”, Computerized alertness test, Bells test, total number of omissions, MoCA, Center of Cancellation | OR 0.47, 95% CI 0.26–0.84 | 0.01 | left lesion laterality |
| Zhou et al. (2004) | asian | 1 | 434 | 47% | 68 |  | LAA,CES | embolic,NS | no | <48h | 3m | MMSE | OR 2.685, 95% CI 1.595-4.521 | 0.000 | left carotid territory infarction |
| **Infarct volume/size** |  |  |  |  |  |  |  |  |  |  |  |  |  |  |  |
| Kulesh et al. (2018) | white | 3 | 57 | 35% | 65 |  | LAA,CES,  SVD,CYP | embolic,  lacunar,NS | NS | 4-21d | 7-14d | MMSE, MoCA, FAB, SVF, Five Word Test, CDT, Schulte Test | NS | <0.05 | Lesion size |
| Prodjohardjono et al. (2020) | asian | 1 | 56 | 38% | 61 |  | NS | embolic,NS | yes | 5d | 3m | MoCA | OR 7.71, 95% CI 1.39–42.91 | 0.019 | Infarct volume (≥0.054 ml) |
| Schellhorn et al. (2021) | white | 1 | 231 | 44% | 72 | Nor-COAST | LAA,CES,  SVD,CYP,  OTH,HS | embolic,  lacunar,NS | no | <1w | 3m | DSM-V | OR 1.04, 95% CI 1.01-1.06 | 0.001 | stroke lesion volume |
| Esmael et al. (2021) | arabic | 1 | 150 | 47% | 62 |  | NS | territorial,NS | yes | <2d,7d | 3m | MoCA | OR 2.01 | 0.005 | ASPECTS score |
| Gu et al. (2022) | asian | 1 | 123 | 38% | 67 |  | HS |  | NS | At admission | 6m | MoCA | OR 3.550, 95% CI 1.866–6.752 | <0.001 | Hematoma Volume |
| Biesbroek et al. (2022) | white,asian | 1 | 2341 | 40% | 67 | Meta VCI Map | NS | embolic,NS | no | <12m | <3m,3-12m,12-24m,>24m | MoCA, NS | OR 1.10, 95% CI 1.05–1.14 | <0.001 | Total infarct volume |
| Ji et al. (2023) | asian | 3 | 300 | 34% | ≤45 years |  | HS,NS | lacunar,NS | yes | NS | <1w | MMSE | OR 8.03, 95% CI 2.28-28.36 | <0.05 | lesion volume >3.00 cm3 |
| Ma et al. (2023) | asian | 1 | 120 | 45% | 63 |  | NS | NS | no | <14d | <14d | MoCA | OR 9.189, 95% CI 2.515–33.568 | 0.001 | Large-area infarction |
| Hobden et al. (2023) | white | 1 | 411 | 47% | 76.16 (median) |  | HS,NS | NS | no | At hospitalization | 195d (183d-210d) | OCS or BCoS | B-coefficient 0.00, 95% CI 0.00-0.00 | <0.001 | larger lesion volume |
| Sachdev et al. (2006) | white | 3 | 169 | 39% | 72 | sydney stroke cohort | LAA,CES,  SVD | embolic,  lacunar | no | <1w | 3-6m | WMS-R, DST, WAIS-R, BNT, TMT-A/B, SDMT, WAB, copying simple figures, finger gnosis and stereognosis, Color Form Sorting Text, FAS, AFT | OR 1.71, 95% CI 1.183– 2.49 | 0.004 | total infarct volume |
|  |  |  |  |  |  |  |  |  |  |  |  |  |  |  |  |
| **Number of acute infarcts/lacunes** |  |  |  |  |  |  |  |  |  |  |  |  |  |  |  |
| Pavlovic et al. (2014) | white | 1 | 294 | 46% | 62 |  | SVD | lacunar | yes | 1-3m | 3-5y | NINDS-AIREN | HR 3.06, 95% CI 1.71-5.50 | <0.001 | number of lacunar infarcts |
| Benjamin et al. (2018) | white | 1 | 120 | 35% | 70 | SCANS | SVD | lacunar | no | >3m,1y,2y,3y | 5y | VFT, M-WCST, BMIPB Speed of Information Processing, DSST, Grooved Pegboard Task, DGS, WMS-III Logical Memory, WMS-III Visual Reproduction, WASI Block Design, WASI Matrix Reasoning, WASI Vocabulary, WASI Similarities | β-coefficient −6.4×10^−2, 95%CI −1.1×10^−1- (−1.8×10^−1) | NS | number of lacunes |
| Ding et al. (2019) | asian | 1 | 145 | 29% | median |  | LAA,CES,  SVD,CYP,  OTH | embolic,  territorial,  lacunar | no | <7d | 6-12m | MMSE, MoCA | OR 1.766, 95% CI 1.243-2.510 | 0.001 | number of acute nonlacunar infarcts |
| Lawrence et al. (2013) | white | 3 | 178 | 37% | 70 | SCANS | SVD | lacunar | NS | >3m | >3m | VFT, M-WCST, BMIPB Speed of Information Processing, DSST, Grooved Pegboard Task | β-coefficient −0.227 (executive function), β-coefficient -0.244 (processing speed) | 0.019 (executive function), 0.006 (processing speed) | Lacunar Infarct Count |
| Williams et al. (2017) | white | 1 | 98 | 34% | 69 | SCANS | SVD | lacunar | NS | >3m,1y,2y,3y | >3m,1y,2y,3y | TMT, Letter Fluency, M-WCST | β-coefficient −0.544, SE 0.175 (executive function) | 0.002 | New Lacunes |
|  |  |  |  |  |  |  |  |  |  |  |  |  |  |  |  |
| **Microbleeds** |  |  |  |  |  |  |  |  |  |  |  |  |  |  |  |
| Chi et al. (2020) | asian | 1 | 65 | 28% | NS |  | LAA,SVD,  CYP | embolic,  lacunar,NS | NS | <7d | 1y | MoCA | OR 6.23, 95% CI 1.34–28.94 | 0.02 | lobar microbleeds |
| Cao et al. (2016) | asian | 3 | 55 | 27% | 67 |  | SVD | lacunar | NS | 3-4m | 3-4m | TMT, SCWT, CFT | β-coefficient -0.352 (attention/executive function) | <0.01 | deep CMBs numbers |
| Yatawara et al. (2020) | asian | 1 | 346 | 40% | 62 | 2 hospitals | NS | NS | yes | At admission | 3-6m | NINDS-AIREN, MoCA | OR 2.50, 95% CI 1.02-4.74 | 0.04 | mixed microbleeds (lobar and deep) |
| Yatawara et al. (2020) | asian | 1 | 346 | 40% | 62 | 2 hospitals | NS | NS | yes | At admission | 3-6m | NINDS-AIREN, MoCA | OR 3.79, 95% CI 1.38-7.86 | 0.00 | three or more mixed microbleeds |
| Nannoni et al. (2021) | white | 1 | 688 | 36% | 65 |  | SVD | lacunar | no | <1y | median 37d | BMET | OR 1.48, 95% CI 1.01–2.18 | 0.047 | Presence ≥ 1 CMB(s) |
| Nannoni et al. (2021) | white | 1 | 688 | 36% | 65 |  | SVD | lacunar | no | <1y | median 37d | BMET | OR 1.43, 95% CI 1.15–1.79 | 0.001 | CMBs total count |
| Nannoni et al. (2021) | white | 1 | 688 | 36% | 65 |  | SVD | lacunar | no | <1y | median 37d | BMET | OR 3.18, 95% CI 1.78–5.68 | <0.001 | Presence ≥ 5 CMBs |
| Nannoni et al. (2021) | white | 1 | 688 | 36% | 65 |  | SVD | lacunar | no | <1y | median 37d | BMET | OR 2.73, 95% CI 1.03–7.27 | 0.044 | Subcortical CMBs |
|  |  |  |  |  |  |  |  |  |  |  |  |  |  |  |  |
| **Brain atrophy** |  |  |  |  |  |  |  |  |  |  |  |  |  |  |  |
| Huang et al. (2020) | asian | 3 | 63 | 25% | 64 |  | HS,NS | NS | yes | 3m | 3m | NS | r = -0.38 | <0.01 | cortical atrophy/thickness |
| Firbank et al. (2011) | white | 1 | 106 | 54% | 80 | stroke registers from seven hospitals | NS | embolic,  territorial,  lacunar | no | 3m | y to dementia | DSM-IIIR | HR 1.32, 95% CI 1.03-1.75 | 0.049 | MTA |
| Lawrence et al. (2013) | white | 3 | 178 | 37% | 70 | SCANS | SVD | lacunar | NS | >3m | >3m | BMIPB Speed of Information Processing, DSST, Grooved Pegboard Task | β-coefficient 0.270 (processing speed) | 0.0026 | Normalised Brain Volume (NBV) |
| Yang et al. (2014) | asian | 1 | 1013 | 44% | 69 |  | LAA,CES,SVD,OTH,  HS,**(TIA)** | embolic,  lacunar,NS | no | <1w of admission | 3-6m | MoCA, MMSE | OR 2.235, 95% CI 1.247-4.005 | 0.007 | MTLA |
| Akinyemi (2015) | black | 3 | 58 | 48% | 60 | two referral hospitals | HS,NS | embolic,  territorial,  lacunar | no | 3m | 3m | CSID - cognitive part, MMSE, vascular neuropsychological battery | OR 2.25, 95% CI 1.16-4.35 | 0.016 | MTA |
| Chen et al. (2016) | asian | 1 | 56 | 38% | 64 |  | LAA,CES,  SVD,CYP | embolic,  lacunar,NS | yes | At admission | 3-12m | 60-min Chinese version of NINDS-CSN, MMSE, MoCA | OR 5.730, 95% CI 1.128-29.101 | 0.035 | global cortical atrophy |

| Arba et al. (2016) | white | 1 | 234 | 38% | 66 | VISTA | SVD,NS,  **(TIA)** | lacunar,NS | NS | NS | 1y | MMSE | PSCI OR 1.94, 95% CI 1.28-2.94; PSD OR 2.31, 95% CI 1.48-3.62 | <0.05 | MTA |
| --- | --- | --- | --- | --- | --- | --- | --- | --- | --- | --- | --- | --- | --- | --- | --- |
| Lim et al. (2018) | asian | 1 | 375 | 41% | 69 |  | NS | embolic,NS | no | <1w | median 153d | NS | r = -0.94 | <0.01 | MTLA grades 3-4 |
| Hagberg et al. (2019) | white | 1 | 109 | 60% | 75 |  | LAA,CES,  SVD,CYP,  HS,**(TIA)** | embolic,  lacunar,NS | yes | At admission,12m,7y | 12m | MMSE, CDT, TMT-A/B, 10-word memory test | OR 0.55, 95% CI 0.35-0.85 | 0.008 | MTLA |
| Molad et al. (2019) | white,arabic | 1 | 397 | 43% | 66 |  | LAA,CES,  SVD,CYP,  OTH,**(TIA)** | embolic,  lacunar,NS | yes | <7d | 2y | MoCA, NeuroTrax computerized cognitive testing (NeuroTrax Corp., Bellaire, TX) | HR 2.37, 95% CI 1.07–5.26 | 0.034 | lower Gray matter volume |
| Molad et al. (2019) | white,arabic | 1 | 397 | 43% | 66 |  | LAA,CES,  SVD,CYP,  OTH,**(TIA)** | embolic,  lacunar,NS | yes | <7d | 2y | MoCA, NeuroTrax computerized cognitive testing (NeuroTrax Corp., Bellaire, TX) | HR 2.21, 95% CI 1.12–4.34 | 0.022 | high Cerebrospinal fluid volume |
| Molad et al. (2019) | white,arabic | 1 | 397 | 43% | 66 |  | LAA,CES,  SVD,CYP,  OTH,**(TIA)** | embolic,  lacunar,NS | yes | <7d | 2y | MoCA, NeuroTrax computerized cognitive testing (NeuroTrax Corp., Bellaire, TX) | HR 2.22, 95% CI 1.14–4.32 | 0.019 | lower Frontal cortex thickness |
| Takahashi et al. (2019) | asian | 3 | 69 | 35% | 73 |  | LAA,CES,  SVD,CYP | embolic,  lacunar,NS | no | <14d | <14d | MoCA | OR 5.3, 95% CI 1.0–27.5 | 0.045 | MTLA |
| Ling et al. (2020) | asian | 1 | 93 | 33% | 70 |  | NS | NS | no | <1w of admission | 3m | MoCA | OR 3.663, 95% CI 1.181–11.359 | 0.025 | Brain atrophy |
| Yang et al. (2020) | asian | 3 | 86 | 33% | 58 |  | NS,**(TIA)** | NS | no | ≤7d | 10d ± 3.4d | MoCA-B | OR 4.673, 95% CI 1.149-19.006 | 0.031 | GCA |
| Schellhorn et al. (2021) | white | 1 | 231 | 44% | 72 | Nor-COAST | LAA,CES,  SVD,CYP,  OTH,HS | embolic,  lacunar,NS | no | <1w | 3m | DSM-V | OR 1.95, 95% CI 1.12-3.41 | 0.019 | MTA pathology |
| Hobden et al. (2023) | white | 1 | 411 | 47% | 76.16 (median) |  | HS,NS | NS | no | At hospitalization | 3d (2.00d-5.00d) | OCS or BCoS | B-coefficient 0.01, 95% CI 0.00-0.01 | <0.001 | total GCA score (cortical atrophy) |
| Sachdev et al. (2004) | white | 1 | 97 | 39% | 72 | 2 hospitals | NS,**(TIA)** | NS | no | 3-6m | 3-6m | TMT-A/B, SDMT, VRI, VRII, block design | r = -0.25 | 0.01 | Cortical Atrophy |
|  |  |  |  |  |  |  |  |  |  |  |  |  |  |  |  |

| **Secondary atrophy** |  |  |  |  |  |  |  |  |  |  |  |  |  |  |  |
| --- | --- | --- | --- | --- | --- | --- | --- | --- | --- | --- | --- | --- | --- | --- | --- |
| Delattre et al. (2017) | white | 1 | 90 | 42% | 63 | 2 hospitals | NS | NS | yes | 6m | 6m | NS | NS | <0.05 | deformation of the left hippocampus |
| Delattre et al. (2017) | white | 1 | 90 | 42% | 63 | 2 hospitals | NS | NS | yes | 6m | 6m | NS | NS | left: p <0.001, right: p <0.05 | reduction of Entorhinal Cortex Area |
| Khlif et al. (2021) | white | 2 | 120 | 68% | 67 | CANVAS | NS | embolic,  territorial,  lacunar | yes | <6w,3m,3y | 3m,3y | HVLT-R | r = 0.29 (delayed recall at 3m), r = 0.31 (immediate recall at 3y), r = 0.34 (delayed recall at 3y) | <0.05 (3m), <0.01 (3y) | lower volume of left hippocampus |
| Aamodt et al. (2022) | white | 1 | 244 | 56% | 72 | Nor-COAST | HS,NS | NS | NS | 2–7d,18m,36m | 36m | MoCA | β-coefficient -41.50, 95% CI -71.25-(-11.75) | 0.006 (significant <0.01) | thinner medial temporal lobe after a left-sided stroke |
| Geng et al. (2022) | white | 3 | 55 | 44% | 64 | 2 hospitals | LAA,CES,  SVD,CYP | embolic,  lacunar,NS | NS | 3-42m | 3-42m | MoCA | β-coefficient 4.117, 95% CI 2.895-1300.565 | 0.008 | left thalamic volume |
| Zuo et al. (2023) | asian | 2 | 52 | 23% | 52 |  | NS | NS | yes | <7d | <10d | TMT-A/B, Modified Chinese version of SCWT | β-coefficient 0.333, 95% CI 0.000–0.010 (executive function) | 0.036 | lower right hippocampus volume |
| Stebbins et al. (2008) | white | 3 | 91 | 49% | 65 |  | LAA,CES,  SVD,OTH | embolic,  lacunar,NS | no | 3-6m | 3-6m | BDAE Commands subtest, Controlled Learning and Enhanced Recall Immediate and Delayed, Self-Ordered Pointing Task, WMS-III Paragraph I and DGS subtests and Form I Mental Control, SDMT, portions of the Behavioral Dyscontrol Scale, MMSE, Figural Recognition Test, CERAD BNT and VFT, Brief version of the RPM, Grooved Pegboard Test | NS | 0.01 | Gray Matter Volume in the Thalamus |

| **WMC (Hyperintensities/**  **Lesions/Diffusion)** |  |  |  |  |  |  |  |  |  |  |  |  |  |  |  |
| --- | --- | --- | --- | --- | --- | --- | --- | --- | --- | --- | --- | --- | --- | --- | --- |
| Wu et al. (2020) | asian | 1 | 487 | 51% | 72 |  | NS | NS | no | At admission | 3y | DSM-IV | OR 3.155, 95% CI 1.868-5.324 | 0.001 | WML |
| Ihle-Hansen et al. (2012) | white | 1 | 182 | NS | NS |  | LAA,CES,  SVD,CYP,  HS,**(TIA)** | embolic,  lacunar,NS | yes | 12m | 12m | TMT-B | β-coefficient 1.75 (executive function) | 0.029 | WML |
| Tu et al. (2013) | asian | 3 | 689 | 41% | 69 |  | NS | NS | NS | >3m | >3m | NINDS, AIREN clinical criteria | OR 2.084, 95% CI 1.507–2.881 | <0.001 | paraventricular WMH |
| Yang et al. (2014) | asian | 1 | 1013 | 44% | 69 |  | LAA,CES,  SVD,OTH,  HS | embolic,  lacunar,NS | no | <1w of admission | 3-6m | MoCA, MMSE | OR 1.083, 95% CI 1.015-1.155 | 0.016 | WMC |
| Chaudhari et al. (2014) | indian | 1 | 102 | 26% | 59 |  | LAA,CES,  CYP,HS | embolic,  lacunar,NS | no | At admission | 6m | criteria of the Canadian Study of Health and Aging (VCI-ND), NINDS–AIREN (VaD) | OR 1.332, 95% CI 1.082–1.640 | 0.007 | ARWMC |
| Pavlovic et al. (2014) | white | 1 | 294 | 46% | 62 |  | SVD | lacunar | yes | 1-3m | 3-5y | NINDS-AIREN | HR 1.42, 95% CI 1.01-2.00 | 0.043 | tARWMC |
| Chen el al. (2015) | white | 1 | 106 | 54% | 80 |  | HS,NS | embolic,  territorial,  lacunar | NS | At admission | y to dementia | DSM-IIIR | HR 1.88, 95% CI 1.05-3.36 | 0.034 | WMH volume |
| Cao et al. (2016) | asian | 3 | 55 | 27% | 67 |  | SVD | lacunar | NS | 3-4m | 3-4m | TMT, SCWT, CFT | β-coefficient -0.457 (attention/executive function) | <0.01 | periventricular white matter disintegrity |
| Makin et al. (2018) | white | 1 | 157 | 41% | 66 (median) |  | NS | embolic,  lacunar | no | At admission | 1y | ACE-R | OR 1.58, 95% CI 1.05-2.44 | 0.03 | Total Fazekas score |
| Zhao et al. (2018) | asian | 1 | 76 | 42% | 66 |  | LAA,CES,  SVD,OTH | embolic,  lacunar,NS | yes | <1w of admission | 1y | MoCA | NS | <0.05 | voxelwise WMH clusters (corpus callosum, corona radiata, and posterior thalamic radiation) |
| Ding et al. (2019) | asian | 1 | 145 | 29% | median |  | LAA,CES,  SVD,CYP,  OTH | embolic,  territorial,  lacunar | no | <7d | 6-12m | MMSE, MoCA, or a neuropsychological battery | OR 3.501, 95% CI 1.652-7.417 | 0.001 | periventricular hyperintensity grading |
| Molad et al. (2019) | white,arabic | 1 | 397 | 43% | 66 |  | LAA,CES,  SVD,CYP,  OTH,**(TIA)** | embolic,  lacunar,NS | yes | <7d | 2y | MoCA, NeuroTrax computerized cognitive testing (NeuroTrax Corp., Bellaire, TX) | HR 2.65, 95% CI 1.15–6.11 | 0.022 | WMH volume |
| Lu et al. (2019) | asian | 1 | 213 | 35% | 65 |  | LAA,CES,  SVD,OTH | embolic,  lacunar,NS | yes | <8d | 3m | MoCA | OR 1.648, 95% CI 1.239-2.191 | 0.001 | Fazekas scale of leukoaraiosis |
| Ling et al. (2020) | asian | 1 | 93 | 33% | 70 |  | NS | NS | no | <1w of admission | 3m | MoCA | OR 8.780, 95% CI 1.210–63.729 | 0.032 | leukoaraiosis |

| Zhao et al. (2021) | asian | 3 | 40 | 18% | 53 |  | NS | NS | yes | <10d | <10d | MoCA | r = 0.549 | <0.001 | total distortion in the orientation of peri-infarct white matter fiber |
| --- | --- | --- | --- | --- | --- | --- | --- | --- | --- | --- | --- | --- | --- | --- | --- |
| Lopes et al. (2021) | white | 1 | 72 | 39% | 62 | STROKDEM | NS | NS | yes | 6.8m ± 1.1m | 6m | TMT-A/B, version of the ST, “code” subtest from WAIS III, Free and Cued Selective Reminding Test, delayed recall score from ROCF, DO 80, semantic AFT, phonemic fluency (p words) test, incomplete letter and number location subtests, ROCF | NS | NS | WMH volume |
| Schellhorn et al. (2021) | white | 1 | 231 | 44% | 72 | Nor-COAST | LAA,CES,  SVD,CYP,  OTH,HS | embolic,  lacunar,NS | no | <1w | 3m | DSM-V | OR 2.73, 95% CI 1.56-4.77 | 0.001 | WMH |
| Schellhorn et al. (2021) | white | 1 | 231 | 44% | 72 | Nor-COAST | LAA,CES,  SVD,CYP,  OTH,HS | embolic,  lacunar,NS | no | <1w | 3m | DSM-V | OR 2.54, 95% CI 1.33-4.84 | 0.005 | WMH |
| Ohlmeier et al. (2022) | white | 2 | 1337 | 41% | 65 | DNA Lacunar 2 Study | SVD | lacunar | no | <1y (median 2d) | median 37d | BMET | OR 1.46, 95% CI 1.24–1.72 | 0.001 | WMH |
| Egorova-Brumley et al. (2022) | white | 3 | 72 | 69% | 67 | CANVAS | NS | NS | NS | 3m | 3m | semantic AFT | NS (semantic fluency) | <0.05 | lower fibre density in 8 left-lateralised tracts (arcuate fasciculus, inferior cerebellar peduncle, inferior occipito-frontal fasciculus, inferior longitudinal fasciculus, optic radiation, superior longitudinal fasciculus III, striato-occipital, and thalamo-occipital tracts |
| Zhao et al. (2022) | asian | 2 | 180 | 43% | 68 |  | HS,NS | NS | yes | 3-12m | 3-12m | MoCA, NS | OR 1.549, 95% CI 1.072–2.240 | 0.02 | lower White Matter Volume (WMV) |
| Zhao et al. (2022) | asian | 2 | 180 | 43% | 68 |  | HS,NS | NS | yes | 3-12m | 3-12m | MoCA, NS | OR 5.199, 95% CI 1.261–21.44 | 0.023 | WMH |
| Zhao et al. (2022) | asian | 2 | 180 | 43% | 68 |  | HS,NS | NS | yes | 3-12m | 3-12m | MoCA, NS | OR 4.631, 95% CI 2.615–8.203 | ≤0.001 | Fazekas Score |
| Kang et al. (2023) | asian | 1 | 37 | 32% | 72 |  | NS | NS | yes | 3m | 3m,12m | K-VCIHS-NP, MMSE | OR 214.0, 95% CI 2.8–224,228.2 | 0.046 | severe WMH |
| Coenen et al. (2024) | white,asian | 1 | 1568 | 40% | 67 | VCI Map | NS | embolic,NS | no | <15m | <15m | different test for each cohort: TMT-A/B, FAS, Digit Symbol Coding, DSG, Symbol Digit Modalities Test | β-coefficient -0.175, SE 0.065 (attention, executive); β-coefficient -0.197, SE 0.071 (information processing speed) | 0.007 (attention, executive), 0.006 (information processing speed) | WMH left anterior thalamic radiation |
| Coenen et al. (2024) | white,asian | 1 | 1568 | 40% | 67 | VCI Map | NS | embolic,NS | no | <15m | <15m | different test for each cohort: TMT-A/B, FAS, Digit Symbol Coding, DSG, Symbol Digit Modalities Test | β-coefficient -0.132, SE 0.054 (information processing speed) | 0.014 (information processing speed) | WMH forceps major |
| Kandiah et al. (2011) | asian (singapore) | 1 | 145 | 23% | 56 |  | SVD | lacunar | NS | At admission | 3m | MoCA, MMSE, FAB | OR 1.15, 95% CI 1.02–1.29 | 0.006 | tWMH |
| Kandiah et al. (2011) | asian (singapore) | 1 | 145 | 23% | 56 |  | SVD | lacunar | NS | At admission | 3m | MoCA, MMSE, FAB | OR 1.45, 95% CI 1.11–1.89 | 0.002 | DWMH |
| Kandiah et al. (2011) | asian (singapore) | 1 | 145 | 23% | 56 |  | SVD | lacunar | NS | At admission | 3m | MoCA, MMSE, FAB | OR 4.13, 95% CI 1.13–15.18 | 0.033 | Severe WMH (modified Fazekas score ≥5) |
| Sachdev et al. (2004) | white | 1 | 97 | 39% | 72 | 2 hospitals | NS,**(TIA)** | NS | no | 3-6m | 3-6m | TMT-A/B, SDMT, VRI, VRII, block design | r = −0.32 | 0.002 | Total Brain Hyperintensity Scores |
| Sachdev et al. (2004) | white | 1 | 97 | 39% | 72 | 2 hospitals | NS,**(TIA)** | NS | no | 3-6m | 3-6m | TMT-A/B, SDMT, VRI, VRII, block design | r = -0.29 | 0.04 | DWMH score |
| Jokinen et al. (2004) | white | 3 | 323 | 51% | 70 |  | NS | NS | NS | 3m | 3m | TMT-A/B, modified short form of ST, coloured dots section (Stroop dots), M-WCST, letter generation (letter K) and AFT, DST, WMS-R, FOME, WAIS-R | NS (mental speed, executive functions, memory, and visuospatial functions) | <0.01 | overall degree of WMHs in different locations |
| Lawrence et al. (2013) | white | 3 | 178 | 37% | 70 | SCANS | SVD | lacunar | NS | >3m | >3m | BMIPB Speed of Information Processing, DSST, Grooved Pegboard Task | β-coefficient 0.085 (processing speed) | 0.41 | higher MD in DTI |
| Lawrence et al. (2013) | white | 3 | 178 | 37% | 70 | SCANS | SVD | lacunar | NS | >3m | >3m | VFT, M-WCST | β-coefficient 0.211 (executive function) | 0.046 | higher RD in DTI |
| Reijmer et al. (2013) | white | 3 | 17 | 43% | NS |  | SVD,NS,  **(TIA)** | lacunar,NS | NS | NS | NS | CFT, SCWT | r = 0.6-0.8 (executive function, information processing speed) | <0.05 | degree of remote secondary WM abnormalities |
| Williams et al. (2017) | white | 1 | 98 | 34% | 69 | SCANS | SVD | lacunar | NS | >3m,1year,2years,3years | >3m,1y,2y,3y | TMT, Letter Fluency, M-WCST | β-coefficient -0.036, SE 0.011 (executive function) | <0.001 (executive function) | DSEG θ |
| Kulesh et al. (2018) | white | 3 | 57 | 35% | 65 |  | LAA,CES,  SVD,CYP | embolic,  lacunar,NS | NS | 4-21d | 7-14d | MMSE, MoCA, FAB, SVF, Five Word Test, CDT, Schulte Test | NS | <0.05 (each) | lower FA in: ipsilateral Thalamus, Anterior Limb of Internal Capsule (Contralateral), Posterior Cingulum (Contralateral) |
| Williams et al. (2019) | white | 1 | 99 | 34% | 68 | SCANS | SVD | lacunar | NS | >3m,1y,2y,3y | >3m,1y,2y,3y,4y,5y | DSM-V, MMSE | HR 3.331, 95% CI 2.076–5.343 | <0.001 | Baseline DSEG-θ |
| Williams et al. (2019) | white | 1 | 99 | 34% | 68 | SCANS | SVD | lacunar | NS | >3m,1y,2y,3y | >3m,1y,2y,3y,4y,5y | DSM-V, MMSE | HR 3.905, 95% CI 2.076–6.650 | <0.001 | Change in DSEG-θ over 3y |
| Egle et al. (2021) | white | 1 | 121 | 35% | 70 | SCANS | SVD | lacunar | NS | At admission | 5y | DSM-V | HR 2.048, 95% CI 1.438-2.918 | 0.000071 | higher MD median (DTI) |
| Jochems et al. (2022) | white | 1 | 229 | 34% | 66 | Mild Stroke Study 3 | NS | lacunar,NS | NS | <3m,1y later | 1y | MoCA | β-coefficient −0.182, 95% CI −0.308-(−0.056) | 0.005 | higher PSMD |
|  |  |  |  |  |  |  |  |  |  |  |  |  |  |  |  |
| **Grey matter changes** |  |  |  |  |  |  |  |  |  |  |  |  |  |  |  |
| Fernández-Andújar et al. (2014) | white | 3 | 17 | 24% | 62 |  | SVD,CYP | lacunar,  territorial,NS | yes | 3m | 3m | lower letter fluency, semantic AFT | β-coefficient 0.74 (verbal fluency in right thalamus), β-coefficient 0.77 (verbal fluency in left thalamus) | 0.005 (right thalamus), 0.001 (left thalamus) | decreased FA in right thalamus and left thalamus |
| Fernández-Andújar et al. (2014) | white | 3 | 17 | 24% | 62 |  | SVD,CYP | lacunar,  territorial,NS | yes | 3m | 3m | lower letter fluency, semantic AFT | β-coefficient -0.54 (verbal fluency in right thalamus) | 0.04 | increased mean diffusivity (MD) values in right thalamus |
| Sachdev et al. (2004) | white | 1 | 97 | 39% | 72 | 2 hospitals | NS,**(TIA)** | NS | no | 3-6m | 3-6m | TMT-A/B, SDMT, VRI, VRII, block design | r = -0.21 | 0.04 | Subcortical Gray Matter Hyperintensity Scores |
|  |  |  |  |  |  |  |  |  |  |  |  |  |  |  |  |
| **Texture analysis** |  |  |  |  |  |  |  |  |  |  |  |  |  |  |  |
| Betrouni et al. (2019) | white | 1 | 160 | 38% | 64 | STROKDEM | HS,NS | NS | no | <72h,6m,36m | 6m | MoCA | r = 0.35 | 0.005 | Kurtosis right hippocampus, left entorhinal cortex, right entorhinal cortex |
| Betrouni et al. (2019) | white | 1 | 160 | 38% | 64 | STROKDEM | HS,NS | NS | no | <72h,6m,36m | 6m | MMSE | r = 0.30 | 0.004 | Inverse Difference Moment (IDM) in left entorhinal cortex, right entorhinal cortex |
|  |  |  |  |  |  |  |  |  |  |  |  |  |  |  |  |
| **Brain connectivity** |  |  |  |  |  |  |  |  |  |  |  |  |  |  |  |
| Ding et al. (2014) | asian | 2 | 38 | 45% | 63 |  | NS | NS | yes | 5-10d | 3m | MoCA | r^2 = 0.51 | <0.001 | decreased FC in the bilateral medial prefrontal cortex and left hippocampus |
| Biesbroek et al. (2022) | white,asian | 1 | 2341 | 40% | 67 | Meta VCI Map | NS | embolic,NS | no | <12m | <3m,3-12m,12-24m,>24m | MoCA, NS | OR 1.27, 95% CI 1.10–1.46 | <0.001 | Network impact score |
| Min et al. (2022) | asian | 3 | 35 | 29% | 51 |  | NS | NS | yes | At admission | <10d | MoCA | r = −0.405 | 0.001 (compared with MoCA) | high DC of right parahippocampal gyrus region |
| Ferris et al. (2022) | white | 3 | 32 | 31% | 66 |  | HS,NS | NS | NS | >6m (mean 70d) | >6m | TMT-A | β-coefficient 11.282 (processing speed) | <0.001 | ATR |
| Ferris et al. (2022) | white | 3 | 32 | 31% | 66 |  | HS,NS | NS | NS | >6m (mean 70d) | >6m | TMT-B | r^2 = 0.438 (executive function) | <0.001 | combination of ATR and forceps minor DTI metrics |
| Pan et al. (2023) | asian | 1 | 676 | 21% | median | 3 hospitals | NS | NS | yes | <7d | 3m | MoCA | OR 1.38, 95% CI 1.17–1.64 | <0.001 | lesion-induced white matter disconnection, particularly in the left dorsolateral prefrontal cortex, basal ganglia, and thalamus |
|  |  |  |  |  |  |  |  |  |  |  |  |  |  |  |  |
| **Brain metabolism** |  |  |  |  |  |  |  |  |  |  |  |  |  |  |  |
| Lee et al. (2021) | asian | 2 | 97 | 36% | 71 |  | NS | NS | NS | once stabilized after stroke | ≥6m | MMSE, NS | HR 10.12, 95% CI 3.3−31.02 | <0.001 | Metabolic Cognitive Signature Score with FDG-PET |
| Yuan et al. (2021) | asian | 1 | 376 | 52% | 66 | 3 hospitals | NS | NS | no | once stabilized after stroke | admission,1m,2m,3m,4m,5m,6m,7m,8m,9m,10m,11m,12m | MoCA | OR 0.37, 95% CI 0.25–0.72 | NS | Left frontal NAA/Cr ratio in 1H-MRS |
| Yuan et al. (2021) | asian | 1 | 376 | 52% | 66 | 3 hospitals | NS | NS | no | once stabilized after stroke | admission,1m,2m,3m,4m,5m,6m,7m,8m,9m,10m,11m,12m | MoCA | OR 0.28, 95% CI 0.04–0.95 | NS | Left thalamus NAA/Cr ratio in 1H-MRS |
| Yuan et al. (2021) | asian | 1 | 376 | 52% | 66 | 3 hospitals | NS | NS | no | once stabilized after stroke | admission,1m,2m,3m,4m,5m,6m,7m,8m,9m,10m,11m,12m | MoCA | OR 0.74, 95% CI 0.53–0.86 | NS | Left hippocampus NAA/Cr ratio in 1H-MRS |
| Ross et al. (2004) | white | 2 | 106 | 44% | 71 | 2 hospitals | NS,**(TIA)** | embolic,  lacunar,NS | NS | 3m | 3m | WMS-R, DST backwards, WAIS-R, BNT, TMT-A/B, SDMT, copying simple figures, WAB ideomotor apraxia subtest items, finger gnosis and stereognosis, Colour Form Sorting Test, AFT, SVF | Mean ± SD 1.19 ± 0.13 (VaD+VCI) bis 1.28 ± 0.14 (NCI), F = 1.996, d.f. = 8.168 | <0.05 | lower NAA/H2O in the occipito-parietal voxel in in 1H-MRS |
| Huang et al. (2020) | asian | 3 | 63 | 25% | 64 |  | HS,NS | NS | yes | 3m | 3m | NS | r (Total Z-SUM-1 - Total Z-SUM-5) = -0.28-(-0.34) | <0.05 | RA (18F-THK-5351 uptake) |
| Kang et al. (2023) | asian | 1 | 37 | 32% | 72 |  | NS | NS | yes | 3m | 3m,12m | K-VCIHS-NP, MMSE | OR 72.2, 95% CI 3.87–9355.2 | 0.023 | Aβ positivity |
|  |  |  |  |  |  |  |  |  |  |  |  |  |  |  |  |
| **Brain vessels** |  |  |  |  |  |  |  |  |  |  |  |  |  |  |  |
| Yang et al. (2023) | asian | 3 | 103 | 18% | 54 |  | SVD,OTH | NS | yes | <14d | <14d | higher STT-B score | β-coefficient 0.223, 95% CI 0.097–0.349 (executive function and memory) | 0.001 | DMVs (more discontinuous or faintly visible DMVs) |
| Yang et al. (2023) | asian | 3 | 103 | 18% | 54 |  | SVD,OTH | NS | yes | <14d | <14d | higher STT-A score | β-coefficient -0.154, 95% CI -0.307-0.000 (language and attention) | 0.049 | CVs (more dilated or larger veins) |
| Seyman et al. (2023) | white,arabic | 1 | 531 | 41% | 67 |  | LAA,CES,  SVD,CYP,  OTH,**(TIA)** | embolic,  lacunar,NS | yes | <72h | 24m | MoCA, NeuroTrax computerized cognitive testing (NeuroTrax Corp., Bellaire, TX) | OR 1.83, 95% CI 1.01-3.35 | 0.048 | High ICC score |

**Supplementary table 4: Others**

| **Author, year** | **Ethnicitiy** | **Study design** | **Sample size** | **Sex (% female)** | **Age (mean)** | **Consortium name** | **Stroke etiology** | **Pattern Ischemic Stroke** | **First Stroke** | **Biomarker measurement time** | **PSCI evaluation after stroke** | **PSCI testing tool** | **Adjusted effect size for primary outcome (if applicable)** | **p-value** | **Biomarkers** |
| --- | --- | --- | --- | --- | --- | --- | --- | --- | --- | --- | --- | --- | --- | --- | --- |
| **Others** |  |  |  |  |  |  |  |  |  |  |  |  |  |  |  |
| Chi et al. (2020) | asian | 1 | 65 | 28% | NS |  | LAA,SVD,  CYP | embolic,  lacunar,NS | NS | <7d | 1y | MoCA | OR 5.77, 95% CI 1.31–25.41 | 0.02 | CA |
| Lim et al. (2014) | asian | 2 | 104 | 38% | 69 |  | NS | embolic,NS | no | 5-14d | >3m | K-VCIHS-NP | OR 1.83, 95% CI 1.15-2.93 | 0.01 | CHIPS |
| Chander et al. (2017) | asian (singapore) | 2 | 209 | 32% | 62 | 3 datasets from singapore | NS | embolic,NS | no | At admission | 3-6m | MMSE, MoCA | β-coefficients of candidate predictor variables 0.17-1.76, SD 0.22-0.52 | NS | CHANGE risk score |
| Makin et al. (2018) | white | 1 | 157 | 41% | 66 (median) |  | NS | embolic,  lacunar | no | At admission | 1y | ACE-R | OR 1.68, 95% CI 1.05-2.76 | 0.03 | SVD score |
| Zhi et al. (2021) | asian | 1 | 157 | 19% | 65 |  | SVD | lacunar | yes | <7d | 3m | MMSE, TMT-A/B, SCWT, CFT, RAVLT, BNT, ROCF | OR 10.957, 95% CI 1.310-91.649 | 0.01 | mSVD score |
| Sung et al. (2021) | asian | 1 | 112 | 36% | 64.5 (median) |  | LAA,CES,  SVD,CYP | embolic,  lacunar,NS | yes | <7d | <7d,3m,1y | MoCA, WAIS III, WMS III, SVF, WCST, DSM-IV | OR 2.74, 95% CI 1.09–6.86 | 0.032 | mSVD score |
| Zhong et al. (2021) | asian | 1 | 103 | 33% | 57 |  | LAA,CES,  SVD,CYP,  OTH | embolic,NS | no | <7d | 3m | 60-min modified VDB | OR 1.038, 95% CI 1.009–1.067 | 0.009 | CHIPS |
| Aamodt et al. (2022) | white | 1 | 269 | 55% | 71 | Nor-COAST | HS,NS | NS | NS | 2–7d,18m,36m | 3m,18m,36m | TMT-A/B, ten-word memory and recall test of CERAD, COWAT, MoCA, AD-8, GDS, DSM-V | β-coefficient 1.37 | 0.01 | BAG |
| Li et al. (2016) | asian | 1 | 365 | 49% | 65 |  | LAA,CES,  NS | embolic,  territorial,NS | no | At hospitali-zation | 1y | MMSE | OR 2.13, 95% CI 1.57–3.26 | <0.01 | High degree of carotid artery stenosis (>70%) |
| Namgung et al. (2023) | asian | 1 | 126 | 59% | 79 (median) |  | NS | NS | no | At admission | <2w | MoCA | β-coefficient 1.040, SE 0.430 | 0.017 | TMT |
| Kong et al. (2023) | asian | 3 | 46 | 28% | 57 |  | HS,NS | NS | yes | 0.3-6m | 0.3-6m | MMSE | Kendall’s tau (τ) correlation coefficient τ =0.879 (LMS-RMS), τ =0.939 (RMS-RPFC) | <0.05 | FC (functional connectivity) value with fNIRS of LMS-RMS (right- and left motor sense cortex) and RMS-RPFC (right motor sense cortex and right prefrontal lobe) |
| Wang et al. (2024) | asian | 1 | 285 | 29% | 62 |  | LAA,CES,  SVD,CYP,  OTH | embolic,  lacunar,NS | no | <24h of admission | 6-12m | MMSE | OR 2.158, 95% CI 1.205–3.863 | 0.01 | low PNI |

**Supplementary table 5: Circulating biomarkers and genetic biomarkers of PSCI**

| Categories | Biomarkers |
| --- | --- |
| Body-fluid biomarkers (number of studies) |  |
| Blood |  |
| Inflammation & oxidative stress (n = 28) | QUIN/KYNA ratio, QUIN, CRP, IL-6, low BChE Activity, RF, low LXA4, Galectin-3, WBC, Amyloid A, Neutrophil percentage, Neutrophil-lymphocyte ratio (NLR), Neopterin, HK, XA, HK-ratio, PA-ratio, Pyridoxal 5́-phosphate, low Picolinic acid, K/T-ratio, systemic immune-inflammation index (SII), 8-OHdG, MDA, DAO, FIB, low sRAGE, TIMP-1, MMP-9 |
| Blood glucose (Diabetes-associated) (n = 10) | Diabetes mellitus, Prediabetes, MAG in diabetes group, History of diabetes, Diabetes duration, HbA1c, SHR, low TIR, MPBG, MAGE |
| Metabolic (n = 22) | Hcy, hypertension with Hcy, UA, low Thiamine (Vitamin B1), low Folate, low Vitamin B12, MMA, low Triiodothyronine (T3), low T3 (syndrome), Anemia (low Hb), Iron, TMAO, hs-cTnT, RA, low Albumin, LDL/LDL-C, low TG, TG/HDL-C Ratio, Triglyceride-glucose index (TyG), HDL-C, Hyperlipidemia, ApoB/ApoA-I ratio |
| Protein biomarkers (n = 11) | BACE1, Aβ-42, low Aβ 1-42, Endostatin, VEGF, ALP, LMP2, low p-tau181, low Neuroglobin, low mBDNF/BDNF, low sDPP4 |
| Cellular (n = 3) | platelet count, MCV, RDW |
| Brain damage marker (n = 4) | pNfL, HMGB1, ATG-5, CysC |
| Combination Inflammation & Metabolic (n = 1) | RF, MMP-9, tHcy |
| CSF |  |
| Inflammation & oxidative stress (n = 2) | IL-1β, IL-10 |
| Urine |  |
| Metabolic (n = 1) | urine formaldehyd concentration |
| Saliva |  |
| Endocrine (n = 1) | Cortisol |
| Stool |  |
| Inflammation & oxidative stress (n = 1) | abundance of Enterobacteriaceae |
| Genetic biomarkers |  |
| SNP (n = 4) | TC and CC genotype carrying MMP-9 rs3918242, ‘C’ allele of rs4580704 (CLOCK gene)/genotypes with C allele of rs4580704 (CLOCK gene), GCLM –588T allele frequency, ACT-TT genotype/ACT T-Allele |
| Hereditary (n = 1) | APOE ε4 |
| miRNA (n = 5) | Serum miR-132, miR-let-7i, miR-21, miR-200b, miR-511-3p |

**Supplementary table 6: Imaging biomarkers of PSCI**

| Categories | Biomarkers |
| --- | --- |
| Imaging biomarkers |  |
| Infarct location | Strategic brain region infarct (thalamus, basal ganglia, frontotemporal lobes; angular gyrus, caudate, globus pallidus, basal forebrain, cingulate gyrus, genu or anterior limb of internal capsule or hippocampus); Infarcts in right parietal lobe; cortical infarction; frontal subcortical infarcts; territorial infarct |
| Infarct volume/size | Lesion size; Infarct volume (≥0.054 ml); stroke lesion volume; ASPECTS score; Hematoma Volume; Total infarct volume; lesion volume >3.00 cm³; Large-area infarction; larger lesion volume; total infarct volume |
| Stroke laterality | dominant-hemisphere hemorrhage; left hemispheric stroke; right lesion laterality; left carotid territory infarction |
| Number of acute infarcts/lacunes | number of lacunar infarcts; number of lacunes; number of acute nonlacunar infarcts; Lacunar Infarct Count; New Lacunes |
| Microbleeds | lobar microbleeds; deep CMBs numbers; mixed microbleeds (lobar and deep); three or more mixed microbleeds; Presence ≥ 1 CMB(s); CMBs total count; Presence ≥ 5 CMBs; Subcortical CMBs |
| Brain atrophy | cortical atrophy/thickness; MTA; Normalised Brain Volume (NBV); MTLA; global cortical atrophy; MTLA grades 3-4; lower MTLA; lower Gray matter volume; high Cerebrospinal fluid volume; lower Frontal cortex thickness; brain atrophy; GCA; MTA pathology; total GCA score; Cortical Atrophy |
| Secondary atrophy | deformation of the left hippocampus; reduction of Entorhinal Cortex Area; lower volume of left hippocampus; thinner medial temporal lobe after a left-sided stroke; left thalamic volume; lower right hippocampus volume; Gray Matter Volume in the Thalamus |
| WMC (Hyperintensities/Lesions/Diffusion) | WML; (paraventricular) WMH; WMC; (t)ARWMC; WMH volume; WMH left anterior thalamic radiation; WMH forceps major; Fazekas scale of leukoaraiosis; severe WMH (modified Fazekas score ≥5); Total Brain Hyperintensity Scores; DWMH (Scores); overall degree of WMHs in different locations; degree of remote secondary WM abnormalities; periventricular white matter disintegrity; voxelwise WMH clusters (corpus callosum, corona radiata, and posterior thalamic radiation); periventricular hyperintensity grading; changes in peri-infarct white matter fibers (in corpus callosum, bilateral internal capsule, external capsule, forceps major, forceps minor, and corticospinal tract); lower fibre density in 8 left-lateralised tracts (arcuate fasciculus, inferior cerebellar peduncle, inferior occipito-frontal fasciculus, inferior longitudinal fasciculus, optic radiation, superior longitudinal fasciculus III, striato-occipital, and thalamo-occipital tracts); higher MD in DTI; higher RD in DTI; DSEG θ; lower FA in: ipsilateral Thalamus, Anterior Limb of Internal Capsule (Contralateral), Posterior Cingulum (Contralateral); Baseline DSEG-θ; Change in DSEG-θ over 3y; higher MD median (DTI); higher PSMD |
| Gray matter changes | decreased FA in right thalamus and left thalamus, increased mean diffusivity (MD) values in right thalamus; Subcortical Gray Matter Hyperintensity Score |
| Texture analysis | Kurtosis right hippocampus, left entorhinal cortex, right entorhinal cortex; Inverse Difference Moment (IDM) in left entorhinal cortex, right entorhinal cortex |
| Brain connectivity | decreased FC in the bilateral medial prefrontal cortex and left hippocampus; Network impact score; high DC of right parahippocampal gyrus region; ATR; combination of ATR and forceps minor DTI metrics; lesion-induced white matter disconnection, particularly in the left dorsolateral prefrontal cortex, basal ganglia, and thalamus |
| Brain metabolism | Metabolic Cognitive Signature Score with FDG-PET; Left frontal NAA/Cr ratio in 1H-MRS; Left thalamus NAA/Cr ratio in 1H-MRS; Left hippocampus NAA/Cr ratio in 1H-MRS; lower NAA/H2O in the occipito-parietal voxel in 1H-MRS; RA (18F-THK-5351 uptake); Aβ positivity |
| Brain vessels | DMVs (more discontinuous or faintly visible DMVs); CVs (more dilated or larger veins); High ICC score |
